# ngsComposer: an automated pipeline for empirically based NGS data quality filtering

**DOI:** 10.1093/bib/bbab092

**Published:** 2021-04-05

**Authors:** Ryan D Kuster, G Craig Yencho, Bode A Olukolu

**Affiliations:** Department of Entomology and Plant Pathology, University of Tennessee, USA; Department of Horticultural Science, NC State University, USA; Department of Entomology and Plant Pathology, University of Tennessee, USA

**Keywords:** quality score, demultiplexing, buffer sequence, qRRS (quantitative reduced representation sequencing), short-read pre-processing, OmeSeq

## Abstract

Next-generation sequencing (NGS) enables massively parallel acquisition of large-scale omics data; however, objective data quality filtering parameters are lacking. Although a useful metric, evidence reveals that platform-generated Phred values overestimate per-base quality scores. We have developed novel and empirically based algorithms that streamline NGS data quality filtering. The pipeline leverages known sequence motifs to enable empirical estimation of error rates, detection of erroneous base calls and removal of contaminating adapter sequence. The performance of motif-based error detection and quality filtering were further validated with read compression rates as an unbiased metric. Elevated error rates at read ends, where known motifs lie, tracked with propagation of erroneous base calls. Barcode swapping, an inherent problem with pooled libraries, was also effectively mitigated. The ngsComposer pipeline is suitable for various NGS protocols and platforms due to the universal concepts on which the algorithms are based.

## INTRODUCTION

With over a decade of mainstream use in biological research, next-generation sequencing (NGS) is a widely applicable technology. Nevertheless, ambiguity regarding best practices in data preprocessing and quality control remains [1, 2]. Illumina short-read sequencing, the predominantly used platform due to its affordability and high yield, is considered the gold standard for NGS data quality. The sequence reads are often used for error correction of long reads derived from other NGS platforms such as PacBio and Nanopore [3]. However, Illumina short reads regularly contain sequencing errors that can significantly impact inferences [4, 5]. Instrument-derived metrics for evaluating base calling accuracy vary across platforms and analytical thresholds are often subjective. Similarly, elevated error rates at read ends or equipment-generated artifacts (e.g. strings of A or G bases) are not always accounted for [6]. Even at low error rates, applications such as variant calling, *de novo* genome and transcript assembly, and microbial strain-level profiling are sensitive to sequencing errors [7, 8]. For example, infrequent errors are misconstrued as minor alleles during variant calling and can result in false positives and false negatives during microbiome strain-level identification, an analysis that is based on exact sequence matching. Quality-trimming of reads has been reported to have a profound impact on sequence assembly, SNP-calling and gene expression [1].

Drawing biologically accurate inferences from NGS data requires high-quality sequence reads and each dataset requires *ad hoc* filtering based on desired application and library preparation technique. Trimming, demultiplexing, adapter removal, quality threshold filtering, artifact removal and error-correction are common pre-processing steps [9]. Multiple tools exist for each of these steps, each with its own parameters as well as limitations. For example, demultiplexing tools often lack the ability to accurately assign barcodes to pooled samples [10]. Some of these tools lack the ability to handle variable length barcodes, and misassignment of sample identities due to barcode swapping is an unresolved problem acknowledged by both independent research laboratories and Illumina [11, 12]. Adapter removal algorithms vary in sensitivity and searching for variable barcodes in highly multiplexed libraries can be tedious. Error-correction methods assume a high-degree of sequencing depth [13]. Overall, with each tool, the number of individual parameters and the optimal sequential order of their application can have significant impact on type I (false positive) and II (false negative) error rates.

Quality scores (*Q* scores) are a valuable metric for selecting high-quality reads. ASCII-encoded *Q* scores report the per-base probability of miscall based on optical fluorescence profiles measured as *Q* = −10log_10_ (probability) [14]. Typically, reads are processed based on *Q* scores to avoid inclusion of erroneously called bases. Over time, changes in Illumina sequencing platforms have modified the interpretation of *Q* scores and consequently altered the practicality and uniformity of their filtering performance [9, 15]. As the capacity to generate more sequence reads in a single sequencing run, newer platforms now bin *Q* scores for practical reasons such as reducing data storage footprint and allowing for realistic data transfer and processing time of ensuing data. The latest Illumina platform (NovaSeq 6000) has superior sequencing quality and yield but only adopts 4 out of the standard 41 Phred quality scores for binning. In addition to instrumentation, *Q* scores are largely influenced by sample and library preparation [16–18]. Here, independent of library preparation methods, we investigate the ability to empirically improve quality filtering by using known sequence motifs to parse reads. We propose a universal set of best practices for empirical quality filtering, and introduce ngsComposer, a user-directed, fully automated, and modular (walkthrough and walkaway mode) pipeline that prioritizes best practices.

There is a community need for standardized practices in performing data preprocessing that makes few assumptions of *Q* scores, and instead relies on knowledge of library preparation and read sequence composition. We present metrics that highlight the efficacy of filtering reads using known sequence motifs coupled with, and in contrast with, *Q* score filtering. NGS reads from multiple Illumina platforms were measured for alignment accuracy and the fate of these reads under different filtering schemes, including optimal order of tools, was evaluated. Furthermore, we developed a fully automated pipeline that handles highly multiplexed data and allows for motif detection as a means of error/adapter detection and removal.

## MATERIALS AND METHODS

### DNA samples and NGS library preparation protocol

#### Materials

The MiSeq Nano 150-PE and NovaSeq SP 150-PE datasets were derived from the quantitative reduced representation sequencing (OmeSeq/qRRS) of a diverse population of 16 hexaploid sweetpotato MDP accessions. These accessions include NASPOT 10 (Kabode), SPK004 (Kakamega), NASPOT7, NASPOT 5/58, NASPOT 11, New Kawogo, Magabali, Wagabolige, NASPOT 5, NASPOT 1, Ejumula, NK259L, Dimbuka-Bukulula, Resisto, Mugande and Haurmeyano. The HiSeq 2500 high output (150-PE) and rapid run (250-PE) datasets were derived from the whole genome sequencing of a temperate-adapted sweetpotato cultivar (Beauregard) and tropical-adapted sweetpotato accession (Tanzania). DNA was extracted from young leaf tissue based on a modified CTAB DNA extraction method. The quality of the DNA samples resuspended in 1X TE buffer was determined on a 0.7% agarose gel to ensure high molecular weight (HMW) DNA. HMW DNA samples were quantified and normalized to 20 ng/ul using PicoGreen assay, diluting with molecular grade water. All subsequent reactions were performed in NEB CutSmart buffer and all the enzymes used are compatible with this buffer.

#### OmeSeq/qRRS library

HMW DNA was sequentially double digested with NsiI-HF and NlaIII. After digestion with Nsi-HF, 96 forward (P1) barcoded adapters were incorporated into genomic fragments by using the isothermal strand displacement activity of the Bst 2.0 warmstart polymerase. Subsequent digestion with NlaIII followed and the 96 reverse (P2) barcoded adapters were incorporated using Bst 2.0 warmstart polymerase. All combinations of the barcoded adapters, resulting in 9216-plex (96 x 96 dual barcoded), were used to ensure nucleotide diversity along entire length of fragments. Although 9216-plex is always achieved, barcoded adapters were pooled before use depending on the number of samples. Consequently, when samples are less than 9216, each sample is dual indexed multiple times. The variable length barcodes ensure nucleotide diversity at the non-variable restriction sites. Aliquots of samples are pooled into a single tube, cleaned and concentrated using Ampure magbeads, and then size selected with BluePippin for fragments between 300 and 600 bp (construct containing genomic insert and adapters). Using the high-fidelity NEB Phusion polymerase, a PCR amplification was performed at 12 cycles in order to incorporate sequences that bind DNA probes on Illumina flow cells. Libraries were diluted to 10 nmol/l for sequencing on each of the Illumina sequencing platforms. To provide quality control, libraries are evaluated on the Tapestation automated electrophoresis at each step after samples are pooled.

#### OmeSeq/WGS library

Using AluI and HaeIII, a partial digest was performed on the HMW DNA in order to enriched for 250–600 bp fragments (peak intensity at 450 bp). The blunt ends of the WGS samples were A-tailed with Klenow fragment exo-. Similar to the qRRS protocol, the 96 forward (P1) and 96 reverse (P2) barcoded adapters with T overhangs and that are complementary to the A overhang of the A-tailed genomic fragments were ligated to the genomic fragments using T4 DNA ligase. The samples were pooled, cleaned and concentrated using Ampure magbeads, and then a secondary digest was performed to eliminate all possible chimeric fragments. The restriction site at the adapter-genomic fragment conjunction are destroyed (adapters are designed so that restriction sites are not re-constituted), so the desired adapter-genomic fragment constructs remain intact. This design was also implemented for the OmeSeq/qRRS adapters. Size selection, quantitative PCR amplification, quality controls and dilution for sequencing are performed as described for the qRRS protocol.

### ngsComposer modular and standalone tools

**Figure 1:**
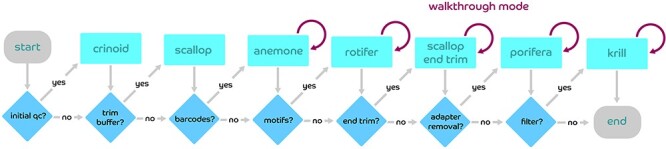
Flowchart of the ngsComposer tools implemented in pipeline mode. Every project directory contains a ‘conf.py’ configuration file that directs the decision at each branch. The optional ‘walkthrough mode’ allows the user to pass new arguments for each tool’s parameters and creates updated *Q* score summary plots concurrently.

The ngsComposer tools are available for standalone use or can be called in order as an automated pipeline using the composer.py (Figure 1).

#### crinoid.py

Crinoid provides summary statistics on *Q* score and nucleotide distribution. Read and quality score lines of a fastq file are traversed *k* bases at a time. Each unique sequence of bases or ASCII scores are stored as a dictionary key. The total number of encounters with that sequence is stored in a list corresponding with the *k* walk position along the read. After all reads have been summarized, the positional information in the dictionary is converted to a matrix of 5 x *n* for nucleotides and 41 x *n* for *Q* scores, where *n* is the maximum read length.

#### scallop.py

Scallop is a simple read trimmer and end-trimming tool. Fixed read positions at the front (−*f*) or back (−*b*) of the read are provided for manual trimming of reads. Users may also opt for quality-based end-trimming using a sliding window approach. In this setting, a window of fixed size (−*w*) walks base-by-base from 3′ to 5′ until the window contains only bases consisting of a given end-trimming *Q* score (−*e*) or higher.

#### anemone.py

Anemone demultiplexes single- or paired-end reads using a tab-separated matrix of barcode and sample names. Reads are first examined for exact matches against the expected set of forward barcodes. To avoid possible *I*/*O* limitations of simultaneous file accession, the corresponding reverse reads are assigned in a separate pass. Although this creates some redundancy in processing, it allows for extreme flexibility in R1/R2 barcoding combinations (e.g. 96 forward and 96 reverse barcoded libraries produced by omeSeq protocol yield 9216 paired output files, 9216 unpaired R1 output files and 9216 unpaired R2 output files). Reads that are not assigned with exact matches (Hamming distance = 0) are optionally subject to further passes through a lenient barcode search with greater leniency in Hamming distance, or mismatch (−*m*). In the instance that multiple barcodes match the queried read, the read is kept as an unknown to avoid sample misassignment.

#### rotifer.py

User-defined lists of sequences are used to search the start of forward and reverse reads for expected motifs. Motifs corresponding to the forward (−*m*1) and reverse (−*m*2) reads are expected in the beginning region of reads due to library construction using restriction enzymes and/or A-tailing of blunt ends. Reads failing to contain these motifs are assumed to begin with sequencing error. Paired-end reads that both pass this test are kept as paired reads, while single ends that pass but that are missing their pair are outputted with a ‘se.’ prefix.

#### porifera.py

A newline separated list of expected adapters (−a1) is provided by the user, optionally containing expected buffer sequences, barcode sequences and known sequence motifs. Adapters are split into substrings of size *k* (−*k*) and each is stored in a dictionary of sequence and distance from the adapter start index. All *k*-mers of distance 0 are scanned for matches within the read, followed by *k*-mers of distance *k*, then 2 *k* and so on. This search process is repeated next with *k*-mers of distance 1, *k* + 1, and repeats for rounds (−*r*) or until all *k*-mers are exhausted. *K*-mer matches pointing to the same start index are assessed per-read until a set of matching positions (−*m*) is reached or the adapter is assumed not to be present. An optional mode (−*t*) allows for a modified Smith-Waterman local alignment to be performed with *t* base overlap to qualify as a hit.

#### krill.py

Integer values for desired *Q* score (−*q*) and percent read composition (−*p*) are provided by the user for threshold filtering. ASCII characters at and above *q* are stored as a list of passing scores. For each read, a failing number of bases is determined by (100 – *p*)*read length. Fastq *Q* scores are then tested 3′ to 5′ for membership in the pass list. If the non-passing characters exceed the failing number of bases, the read is rejected.

### Comparing performance of motif and threshold filtering

For each of the datasets tested (MiSeq Nano, HiSeq 2500 high output 2 x 125, HiSeq 2500 rapid run 2 x 250, NovaSeq 6000), reads were first trimmed to remove buffer sequences (6 bp) and demultiplexed with 1 mismatch using anemone.py. Reads were used as the basis for downstream analysis of motif-detection and threshold filtering tools, as they are expected to begin with the RE digest motif immediately following the barcode sequence.

### Filtering methods

Filtering was performed using threshold-filtering (krill.py) and motif-filtering (rotifer.py). Under threshold filtering, only reads consisting 90% of above of *Q* scores 30 or higher were considered as passing filtering. Motif filtering was performed in a library-specific manner. The HiSeq 2500 datasets were blunt-end fragmented using AluI/HaeIII, followed by A-tailing and adapter ligation. Only HiSeq reads beginning with the sequences ‘TCC’ and ‘TCT’ were considered passing. The MiSeq and NovaSeq datasets were prepared using the Omeseq/qRRS protocol that fragmented the genome with NsiI/NlaIII restriction enzymes. R1 reads in these libraries contained a ‘TGCAT’ motif and R2 reads contained a ‘CATG’ motif. For each dataset, reads were first processed using threshold- and motif-filtering tools with failed reads written separately to file. For each of these output files, all passing and failing reads were processed again using the corresponding tool to be compared. For example, reads passing and failing the krill.py threshold filtering steps were then motif-filtered using rotifer.py, producing four final output files representing all combinations of pass and fail for combinations of the first and second tool.

### Collapsing unique reads

After demultiplexing using variable length barcodes, reads within each library were end-trimmed to identical length before filtering to facilitate unbiased read comparisons downstream. After filtering methods were applied, all sequences were extracted from the fastq output using ‘sed’ commands. An awk command was then applied to sequence files to collapse identical reads while retaining the frequency of the unique reads. The ratio of collapsed reads to the total number of reads was calculated per-file.

### Calculating empirical base-calling error rates in barcode sequences

All forward (R1) reads from the MiSeq, HiSeq 2500 Rapid Run, HiSeq 2500 High Output and NovaSeq 6000 datasets were subjected to multiple rounds of demultiplexing using the ngsComposer tool anemone.py. After first demultiplexing perfect matches, the unassigned reads were subsequently demultiplexed with a Hamming distance of 1. This process was repeated with unknown reads, increasing the mismatch value at each step until reads could no longer be identified having exceeded their inherent minimum Levenshtein distance of 3 (https://github.com/roy-ht/editdistance). For each step, the reads in the resulting output files were compared base-by-base to their corresponding barcode sequence. On a per-position basis, the probability of error was calculated as the number of bases in that position that did not match the assigned barcode divided by the total number of reads assessed at that level of mismatch and fewer. The ASCII-encoded *Q* scores were counted and grouped in the same manner.

### Validating software performance using simulated reads

A set of 998 611 reads were simulated using the first chromosome of the *Ipomoea batatas* assembly (NCBI GenBank assembly accession: GCA_002525835.2). Simulation of restriction enzyme fragmentation was performed using *NsiI* and *NlaIII*, and each fragment was concatenated with variable length adapters containing variable length barcodes (simulating the OmeSeq/qRRS library preparation method). Resulting fragments were drawn randomly from a normal distribution with a mean read length of 400 bp and a standard deviation of 100 bp. The unmutated reads were saved separately alongside the known adapter position, if present. Multiple adapter trimming tools were compared with ngsComposer. A minimum adapter overlap value of length 12 was used as this was found to avoid an overabundance of misassignments and allowed a better unbiased comparison of tools. The standalone version of porifera used adapter validation by supplying the *NsiI* and *NlaIII* sequences and was compared with a pipeline instance of the tool that automatically reduces the search space on a per sample basis.

## RESULTS

### Library design and empirical *Q* score assessment

**Figure 2:**
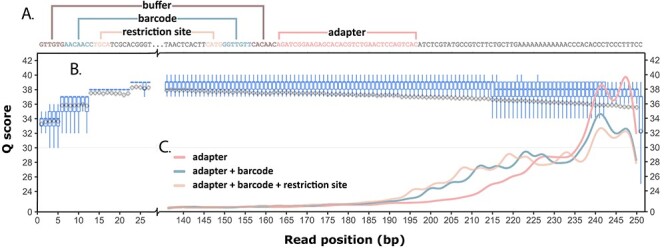
QC plot highlighting impact of adapter removal parameters implemented in ngsComposer. (**A**) Diagram of adapter-contaminated read displaying buffer region, barcode and restriction sites, as well as the corresponding reverse-complement adapter regions. (**B**) Boxplots show the tendency towards lower *Q* scores at 5′ and 3′ ends. Gray diamonds and lines show the mean *Q* score and standard deviation per position. (**C**) Read length density after adapter removal using ngsComposer pipeline. Adapter only (red), adapter through barcode (blue) and adapter through restriction site (yellow) each show different performance in adapter detection.

To assess the effectiveness of *Q* scores compared to motif-detection as a filtering parameter, NGS data from sweetpotato (*Ipomoea batatas*) accessions and cultivars were considered from multiple Illumina platforms, each with distinct profiles. Fastq files originating from various Illumina platforms (i.e. Miseq, HiSeq 2500 Rapid Run, HiSeq 2500 High Output and NovaSeq 6000) represent a variety of read length, dye chemistry and levels of Q score binning (Supplementary Figures S1–S4). All DNA libraries were prepared using custom-designed adapters and enzyme fragmentation. Each of the adapter pairs of the dual-barcoded datasets include a 6 bp buffer sequence region upstream of the barcode sequence, 7–10 bp variable length barcode sequence and a 3–5 bp motif complementary to each of the two restriction cut sites in the insert DNA (Figure 2A). For the whole genome sequencing libraries, a T motif after the barcode is complementary to the A overhang on genomic fragments following A-tailing of blunt ends. By using adapters that contain a fixed-length and high-diversity ‘buffer sequence’ region (as implemented in the OmeSeq protocol), the sequencing-by-synthesis reaction is allowed to stabilize before base-calling in the barcode regions begins. This has a protective effect on the barcode sequences used to determine sample identity, as the initial bases in a sequencing-by-synthesis reaction tend to harbor lower *Q* scores or elevated base calling error (Figure 2B, Supplementary Figures S1–S4) [12, 19]. Fixed-length end-trimming was performed by ‘scallop.py’ in this buffer region before demultiplexing.

**Figure 3:**
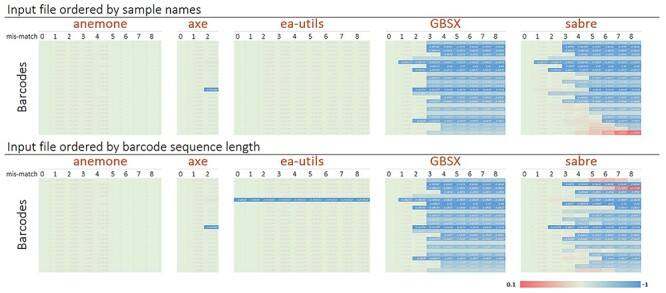
Benchmarking NGS read demultiplexing tools. Several tools are influenced by the order (top and bottom panel) of barcodes when searching for potential matches (ea-utils and sabre). In some instances, tools preferentially reassign reads to a different sample as mismatch is increased across columns from left to right. Values within the heat map indicate the degree of deviation computed as the proportion of mis-assigned reads at mismatch > 0 relative to assignment at a mismatch = 0, which is specified within each tool. The midpoint, green, indicates zero deviation.

Variable length barcodes with a minimum Levenshtein/edit distance of three were implemented to reduce platform-derived phasing error [11, 17]. Using variable length barcodes is also very important for achieving high nucleotide diversity in libraries fragmented with restriction enzymes or that were A-tailed, since the sequence motif region of fragments inherently lack the nucleotide diversity required for optimal run performance and generation of high-quality reads. The tool ‘anemone.py’ was used to demultiplex the libraries using a dual-indexed barcoding scheme, which is known to improve assignment accuracy [12]. Anemone.py prevents sample misassignment in the event that multiple barcodes have equal Hamming distance from a given read. Reads that align to multiple barcodes remain unassigned. Misassignment can be found in several widely used demultiplexing tools, which apply sample identities preferentially to the first indexed barcode when ‘nesting’ occurs (Figure 3).

**Figure 4:**
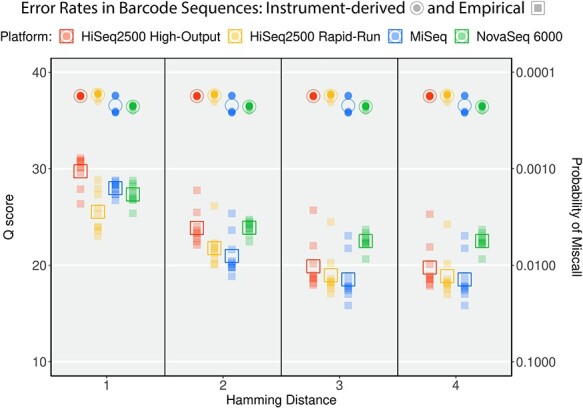
Quality scores from four Illumina platforms measured within barcode regions of reads. The empirical rate of base-calling errors (squares) were sequentially calculated using increasing Hamming distance in the ngsComposer demultiplexing tool, anemone. The *Q* scores for these bases reported by Illumina software reveal underestimation of base-calling error. Open shapes indicate the mean *Q* scores, while solid shapes indicate *Q* scores for individual base positions along the barcode region.

Taking advantage of anemone.py’s false positive sensitivity, we tested the sequencing accuracy of barcode regions by demultiplexing at increasing values of hamming distances. The per-base error rate was calculated alongside the reported instrument-derived per-base *Q* scores (Figure 4). Empirically calculated miscall probabilities were lower than those produced by the sequencing hardware with quality score mean differences across platforms ranging between 7.81 and 18.7 (Figure 4, Supplementary Table S1). These corresponds to approximately 6–74 times increased probability of miscall. These results agree with previous attempts to empirically validate *Q* scores by alignment to known reference genomes [20–23] as well as to barcoding region.

### Evaluating read populations using expected motifs

After barcode removal, we used the presence of error-free restriction enzyme site motifs as an early indicator of read reliability (motif-based error detection and filtering). Reads with error in the motifs were observed to also contain higher error rates along the entire length of the read when compared with the intact motifs (Table 1, Supplementary Figures S1–S4). Reads filtered sequentially using classic *Q* score-dependent threshold-filtering followed by motif-filtering produced populations of passing and failing reads, which were then used to evaluate the performance of both approaches. We expect a high false negative rate for stringent *Q* score threshold filtering and a high false positive rate for relaxed *Q* score threshold filtering. Since the threshold is subjective, there is currently no objective setting for the optimal threshold to minimize these false positive and false negative rates.

**Table 1:** Efficacy of filtering methods using an unbiased estimate, read compression

Illumina platform	Filtering method(s)	Δ Compression^a^ (R1)		Failed (%)^b^	Δ Compression^a^ (R2)		Failed (%)^b^
		Pass	Fail		Pass	Fail	
MiSeq	Q30/P90	3.42	−9.3	12.33	4.54	−10.27	16.1
	Motif	2.48	−5.06/−4.9^c^	7.55	0.88	−3.03/−2.7^c^	2.75
	Q30/P90 + motif	6.06	−4.14	18.92	5.29	−0.7	17.99
HiSeq 2500 (Rapid Run)	Q30/P90	11.08	−40.29	21.16	23.74	−24.36	47.63
	Motif	0.72	−36.63/−32.4^c^	1.06	1.98	−21.69/−19.2^c^	4.78
	Q30/P90 + motif	11.58	−20.24	21.73	25.22	−8.74	49
HiSeq 2500 (High Output)	Q30/P90	5.07	−17.09	22.06	4.4	−24.93	14.65
	Motif	0.45	−30.56/−26.0^c^	1.08	0.73	−41.08/−36.4^c^	1.08
	Q30/P90 + motif	5.46	−20.51	22.53	4.68	−11.84	15.1
NovaSeq 6000	Q30/P90	7.25	−35.9	16.3	10.63	−33.8	23.12
	Motif	1.6	−8.03/−6.6^c^	8	2.89	−2.24/−0.7^c^	21.65
	Q30/P90 + motif	8.59	1.75	22.71	12.43	12.23	38.52

^a^Δ Compression: change in compression rates of filtered reads were normalized against randomly subsampled raw data.

^b^The percentage of raw reads that did not pass the given filtering technique.

^c^Compression rates in reads failing presence of motif were recalculated using only the non-motif portion of the read.

Read compression was applied to the results of filtering as an unbiased determinant of base calling accuracy. Read depth refers to the frequency of reads aligning uniquely to a given reference locus. The restriction enzyme-based qRRS libraries examined in this study consist of numerous, identical fragments of DNA that align flush and perfectly overlap the other allelic sequences originating from the same locus and across samples. Collapsing instances of identical sequence reads within NGS datasets, termed the compression rate, were used as a proxy for unbiased error rate estimates. The compression rate value approximates the average read depth across the genome, i.e. number of times an allele was sequenced. High error rates will increase the generation of new novel reads and consequently lower compression rates. The compression rate was found to be a more sensitive metric for error rate estimation compared to using a reference genome, where alignment scores (e.g. *E*-value) are sensitive to sequence length and other parameters in the alignment algorithm [24].

**Figure 5:**
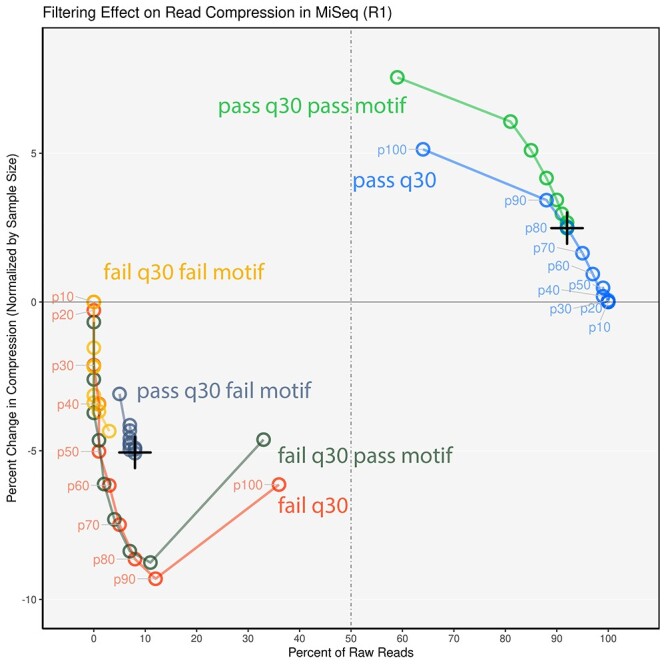
Sample results using *Q* score threshold-filtering and motif-based filtering. The difference in read compression between the subsetted filtered reads was normalized against compression of MiSeq-derived raw data of the same sample size. Reads passing both tools have the highest compression values indicating a reduction in sequencing error. Both filtering methods detect erroneous reads that are non-overlapping, hence, underscoring the need of both strategies.

The motif-based filtering approach complemented the *Q* score threshold-filtering approach. In all datasets analyzed across all NGS sequencing platforms in this study, the highest compression was achieved when both filtering approaches were applied (Figure 5). Read sequences failing these filtering approaches consisted of more unique/singleton reads due to increased error rates and consequently had a negative change in compression when normalized against random sampling of the unfiltered dataset to account for the effect of reduced sample size. These patterns persisted in the failed reads even when limiting the scope to the body of the read while excluding the motif region, further validating that a failure to sequence the motif accurately will lead to miscall within the rest of the read (Table 1). Motif-filtering improvement of compression was found to be most effective in the MiSeq dataset (2.48% improvement alone, 6.06% improvement combined with threshold of q30 at 90%). In some instances, the improvement was marginal (0.45% improvement in HiSeq 2500 high output); however, the number of reads removed using motif-filtering was much lower than with threshold filtering (Table 1). It is also worth noting that the OmeSeq protocol produced raw sequence reads with high-quality, hence, we would typically expect significantly higher improvement with data derived from most library preparation protocols. The subset of reads removed by the two filtering methods had little overlap as each appeared to target nearly distinct reads within the overall dataset.

**Table 2:** Comparison of adapter removal tools on simulated NGS reads. Filtering settings includes using Illumina adapters without (single adapter) and with barcode sequences (96 barcoded adapters) as contaminating sequences

Approach	Tools	Read	False negatives (%)	False positives (reads)	Total user time (seconds)
Single adapter	AdapterRemoval	R1	29.4	3044	13.0
		R2	29.5	3075	
	Cutadapt	R1	28.6	0	24.9
		R2	28.9	0	
	Porifera (standalone)	R1	30.0	2	29.2
		R2	30.5	1	
	Skewer	R1	29.5	0	24.5
		R2	29.7	0	
96 barcoded adapters	AdapterRemoval	R1	11.7	161 932	571.7
		R2	11.3	160 429	
	Cutadapt	R1	12.5	644	2699.4
		R2	12.7	689	
	Porifera (standalone)	R1	15.2	63	543.1
		R2	16.3	4	
	Porifera (ngsComposer)	R1	14.1	4	79.4^a^
		R2	14.5	2	
	Skewer	R1	13.5	5	3050.7
		R2	13.6	107	

^a^Simultaneous run time for R1 and R2 (as part of ngsComposer pipeline).

Note: Analysis of simulated data for all tools was performed on a MacBook Air (8 GB 3733 MHz LPDDR4X, 1.1 GHz Quad-Core Intel Core i5) using a single thread.

### Improved adapter detection using barcode-specific search schema

Adapter removal is an important step in many NGS libraries and detection can be improved using expected motifs. Adapter sequences including barcode and restriction site motifs are expected to increase adapter detection sensitivity as these sequences are further upstream of the characteristic 3′ drop in sequence quality [21, 25]. Herten *et al*. [11] showed the inclusion of restriction motifs improved adapter detection. Here, the tool porifera.py has been developed to k-mer walk through a list of adapters (i.e. combination of adapter, buffer sequences, barcode sequences and known motifs) and search a user-defined number of rounds (or default) until aligned *k*-mers point to the same start index or a read is deemed to be adapter-free. The *k*-mer approach avoids local alignment issues encountered when a string of ‘A’ or ‘G’ sequencing artifacts appear in the instance of a deeply embedded adapter. The ngsComposer pipeline narrows the adapter search space by only attempting to align reads with their associated adapters, which contain sample-specific barcodes. Adapters including barcodes and barcodes with restriction sites detected reads at a higher frequency and removed non-genomic DNA (Figure 2C).

### Validating software performance using simulated reads

Simulation of Illumina reads allowed for testing the performance of the motif-based NGS filtering approach highlighted in the ngsComposer pipeline and tools. We confirmed our findings that a motif-based filtering approach is useful for NGS data filtering, as shown in both real and simulated data (Supplementary Figures S9 and S10). We also confirmed the obvious benefit to including barcodes, a contaminating non-genomic sequence, in the adapter searching step (Table 2, Supplementary Figures S11 and S12). Overall, the false positive rates were similar across all tools except for AdapterRemoval, which had very high false positive rates. While including the barcodes as part of the contaminating adapter sequence should be considered best practice for multiplexed libraries, we evaluated a naïve approach that only uses the Illumina adapter sequence. While using a single adapter sequence also produced low false positives, it led to about twice as much false negative rate (i.e. reduced detection of adapters in reads). The false negative rates were similar across all tools tested, with Cutadapt and AdapterRemoval performing slightly better but typically at the expense of elevated false positive rates. The ngsComposer pipeline leverages speed and accuracy by only searching for those adapters containing the unique barcodes associated with the appropriate sample (Table 2). In pipeline mode, porifera is 7–38 times faster compared to other tools tested. In standalone mode, the speed is similar to AdapterRemoval and 5–5.6 times faster than Cutadapt and Skewer, respectively (Table 2).

### Implementation

The software, ngsComposer, is designed with simplified user input at every critical step in data filtering. Any of the provided tools may be run individually or as an automated pipeline. In pipeline mode, users have the option to see read summaries and qc plots and re-define variables on the fly as a part of ‘walkthrough’ mode. Multiple libraries from different sequencing runs may be combined for preprocessing, each with its own set of barcodes and associated unique sample IDs. In pipeline mode, paired end reads are automatically recognized and pairing preserved throughout. Reads that become unmated due to removal of paired R1 or R2 reads are retained in all subsequent steps in a single end reads directory.

## DISCUSSION

Filtering NGS data are a routine procedure intended to retrieve sequence reads with minimal base calling error, as well as identification and removal of low-quality base calls by trimming reads at the 5′ and 3′ ends. Traditionally, *Q* scores are the exclusive determinant of the reliability of sequencing certainty. While *Q* scores are useful as a filtering metric, there is mounting evidence and suggesting that *Q* scores should not be taken at face value and can have variable interpretations across NGS platforms. The higher empirical estimates of error rates in the barcode and restriction site regions described here imply that *Q* scores may not match the expected logarithmic Phred base-calling error probability. Some SNP calling and filtering approaches have opted to ignore *Q* scores altogether due to a biasing tendency against SNPs on distal ends of reads [26]. Only a few efforts exist that attempt to determine read accuracy with certainty and independent of platform-derived quality scores. Alignment to reference assemblies is possible with smaller genomes but may vary based on reference assembly and the significance of single base mutations may not be captured by alignment scoring penalties alone. Studies reveal that accuracy of various error correction algorithms differed considerably across all types of data set [24].

Our pipeline works off the assumption that reads containing high-quality base calls in expected sequence (barcodes, restriction site and A-tailed reads) near the 5′ end will contain higher quality base-calling across the entire read length and will contain fewer erroneous base calls internal to the read body. This assumption was confirmed by elevated error rates in entire read length whenever there was base calling error in the motif. Emphasizing motif-filtering retains reliable high-quality base-calls required for applications such as variant calling, strain-level microbiome profiling and *de novo* assembly of genomes and transcriptomes. Motif-detection is independent of platform and encompasses some of the errors that might be detected in other filtering approaches.

To provide additional validation for the efficacy of motif-based filtering, we used the read compression rates to confirm that errors in motif region are often tied to additional error elsewhere in the body of the read. Read compression is an unbiased measure of sequencing accuracy. Lower compression rates indicate that at least one base miscall or indel has occurred in the read. An increase in read compression indicates that filtering has recovered high-quality reads and eliminated unique reads derived from erroneous base calls that would normally reduce compression. Not surprisingly, read reliability was most improved by implementing empirical motif-detection alongside machine-generated *Q* score threshold-filtering. In combination with the motif-based approach, *Q* score filtering thresholds can be relaxed to reduce false negative rates without the consequence of increasing false positive rates. While subsets of reads filtered by motif-presence always had an improvement in read compression, these reads were often classified independently from reads containing high *Q* score composition. This underscores the sensitivity of motif-based filtering and utility for identifying false negatives associated with *Q* score-based filtering. Motif inclusion has been a previously subtle feature investigated by Herten *et al*. [11]. It has the potential to bypass platform and even sequencing run variation. It is possible that strict motif-filtering and the compression rate analysis reported here could have applications in reviving the wealth of previously published sequencing data.

The ngsComposer software is a fastq preprocessing pipeline designed for straightforward and repeatable results that extend the power of *Q* score-based filtering. As revealed by the datasets generated with the OmeSeq protocol, reported here, consideration of assay design during library preparation can improve overall sequencing reaction quality and features in library constructs can provide information and quality control that can enhance data preprocessing. Furthermore, ngsComposer provides researchers with a practical framework to reliably handle multi-sample projects with complex barcoding schema and organizing paired-end data from their raw to analysis-ready state.

**Key Points**

- The filtering pipeline, ngsComposer, automates and simplifies NGS data preprocessing while effectively mitigating barcode swapping during demultiplexing.
- Phred-like *Q* scores remain a useful benchmark for filtering, but empirical evidence presented here reveal they tend to be overinflated.
- The use of an empirically based approach that employs known motifs for error detection and filtering yields high-quality sequence reads not detected with *Q* scores alone.
- An objective compression rate metric, presented here, provides a sensitive and unbiased method for estimating error rates within NGS data.
- The pipeline also features an adapter removal tool that is robust for deeply embedded contaminating adapter sequences existing within the context of base calling errors and strings of A and G artifacts.

## Supplementary Material

Supplementary_Kuster_et_al_ngsComposer_bbab092

## Data Availability

All raw NGS data used for this study will be publicly available on the NCBI SRA database. Software used is available at https://github.com/ryandkuster/ngsComposer and all analytical steps are available at https://github.com/ryandkuster/ngsComposer_analysis.

## Notes

**Ryan Kuster** is a PhD student at the Department of Entomology and Plant Pathology, University of Tennessee. His research focuses on integrating molecular and bioinformatic applications for genomic and metagenomic studies.

**Craig Yencho** is a professor at the Department of Horticultural Science, NC State University. He leads global efforts for crop improvement and development of genetics and genomic tools in sweetpotato and potato.

**Bode Olukolu** is an assistant professor at the Department of Entomology and Plant Pathology, University of Tennessee. His research focuses on understanding the genetics of complex traits in maize and polyploid crops.
