# Supplementary material for: ngsComposer: an automated pipeline for empirically based NGS data quality filtering
Source: Brief Bioinform. 2021 Apr 5;22(5):bbab092. doi: 10.1093/bib/bbab092 (PMC8425578; doi:10.1093/bib/bbab092)
Supplement: Supplementary_Kuster_et_al_ngsComposer_bbab092 [file supplementary_kuster_et_al_ngscomposer_bbab092.docx]

MiSeq (R1) – raw

MiSeq (R2) – raw


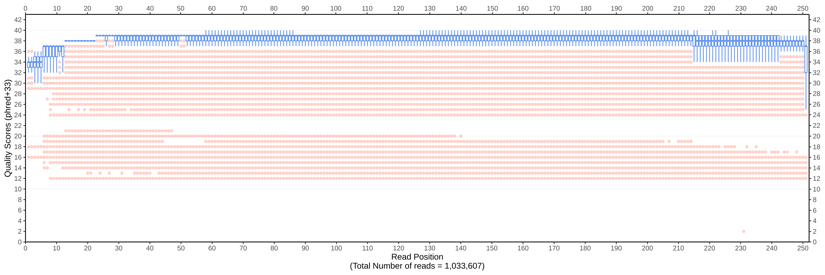

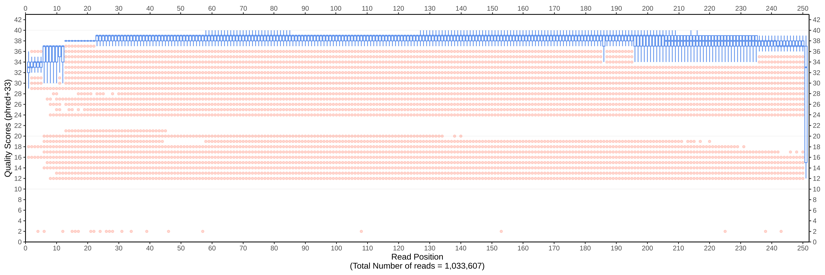


MiSeq (R1) – demultiplexed

MiSeq (R2) – demultiplexed


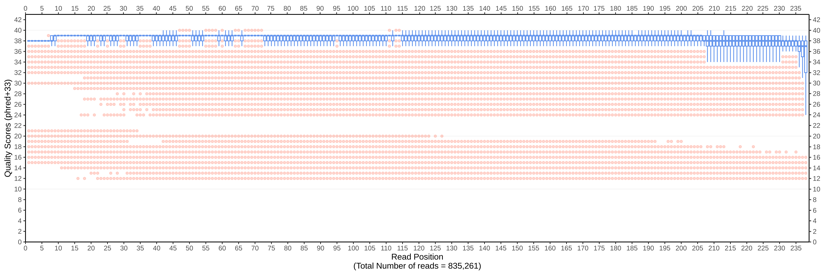

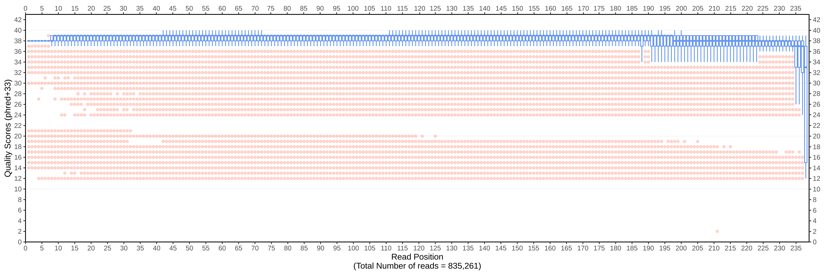


MiSeq (R1) – motif-filtered

MiSeq (R2) – motif-filtered


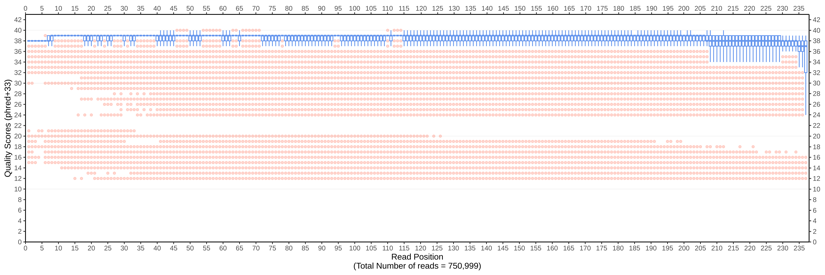

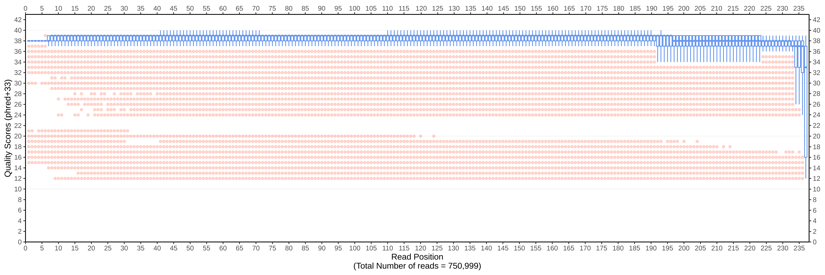


MiSeq (R1) – end-trimmed

MiSeq (R2) – end-trimmed


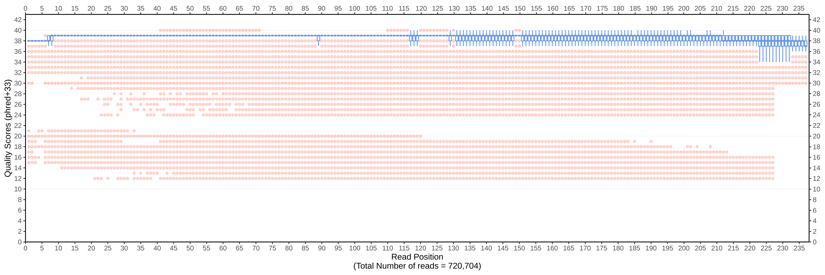

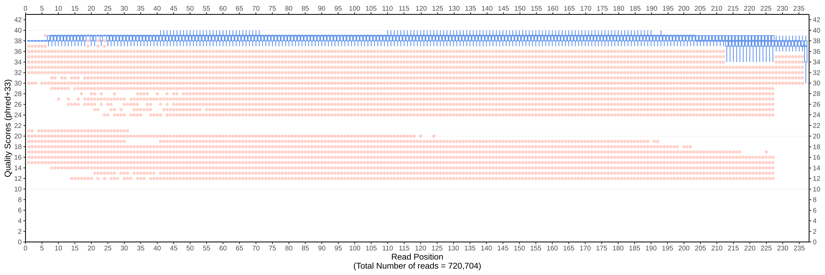


MiSeq (R1) – adapted

MiSeq (R2) – adapted


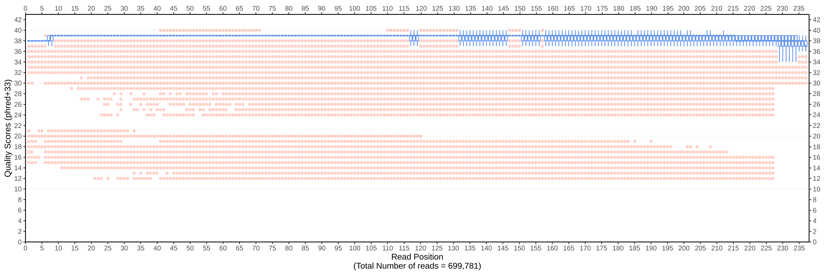

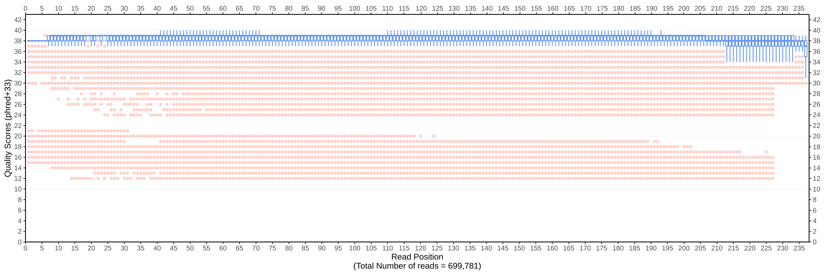


MiSeq (R1) – threshold-filtered

MiSeq (R2) – threshold-filtered


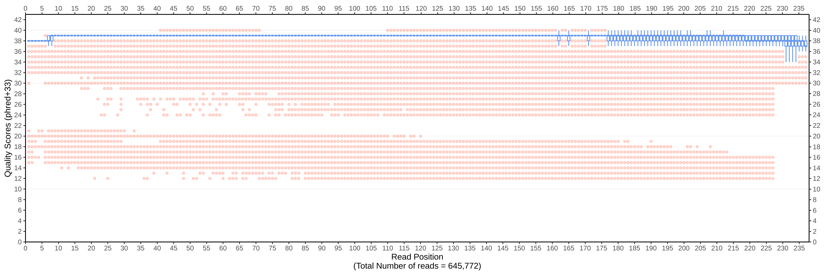

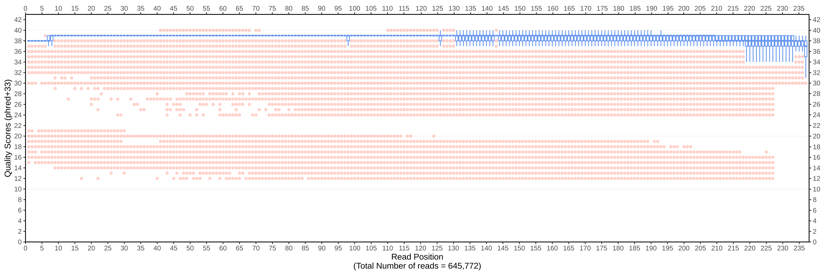


**Figure S1**. Summary (boxplot) of quality scores (Q score) of Miseq Nano paired end reads at each step of the ngsComposer pipeline. Demultiplexing was performed with a maximum mismatch of 1, while the Q score threshold filtering only allowed for reads with at least 90 % of bases in read having a minimum Q score of 30. Red dots indicate outliers, which also indicates the level of quality score binning.

HiSeq 2500 high output (R1) – raw

HiSeq 2500 high output (R2) – raw


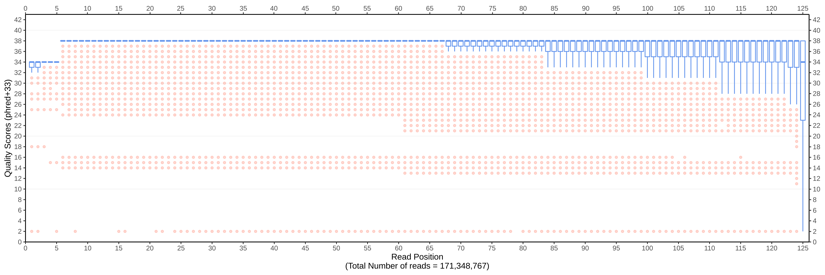

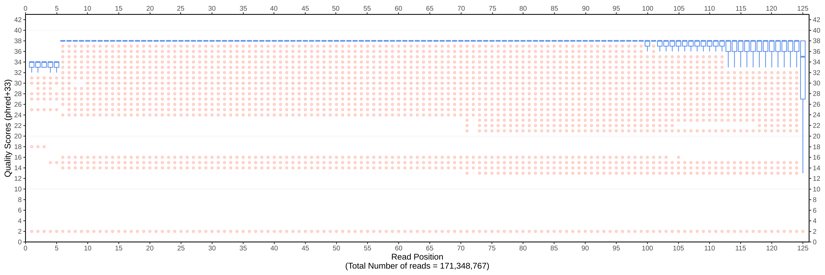


HiSeq 2500 high output (R1) – demultiplexed

HiSeq 2500 high output (R2) – demultiplexed


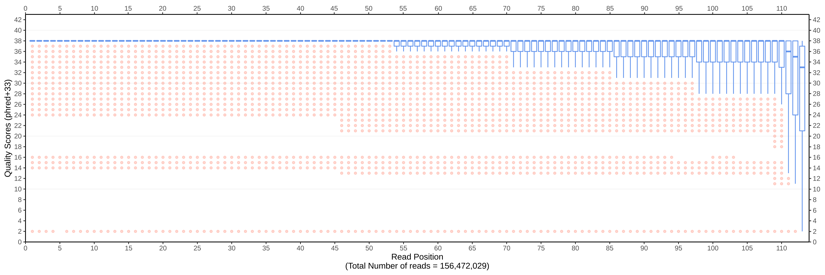

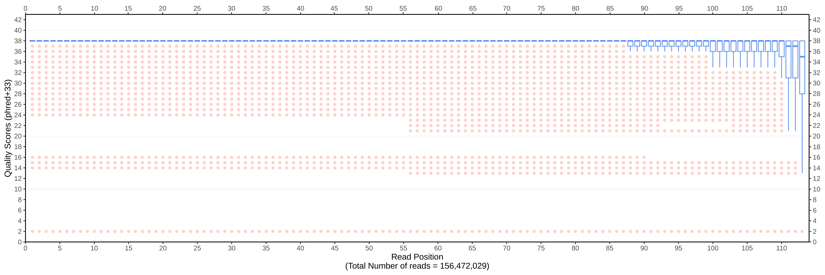


HiSeq 2500 high output (R1) – motif-filtered

HiSeq 2500 high output (R2) – motif-filtered


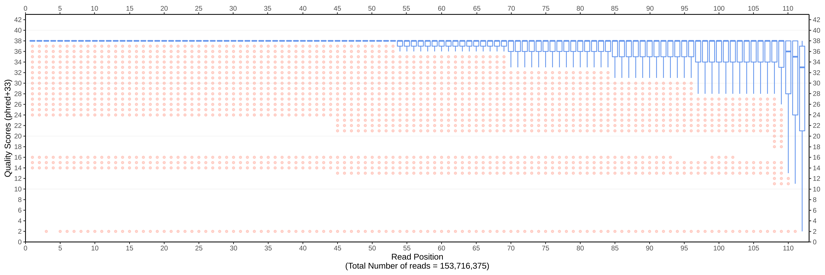

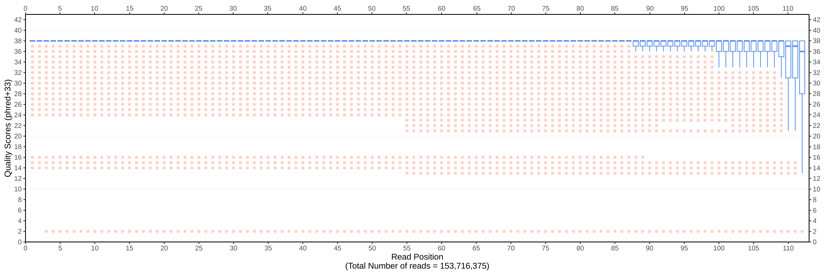


HiSeq 2500 high output (R1) – end-trimmed

HiSeq 2500 high output (R2) – end-trimmed


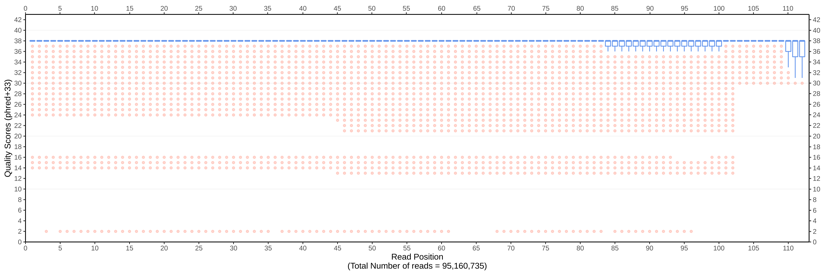

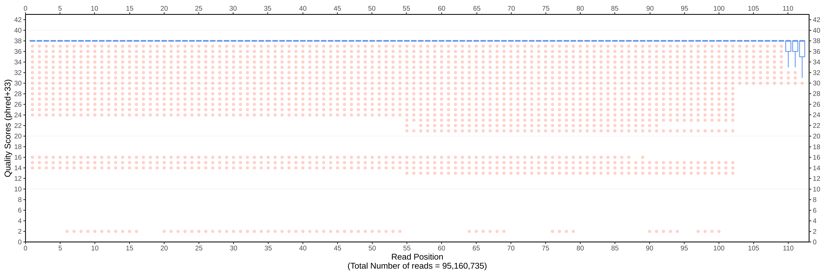


HiSeq 2500 high output (R1) – adapted

HiSeq 2500 high output (R2) – adapted


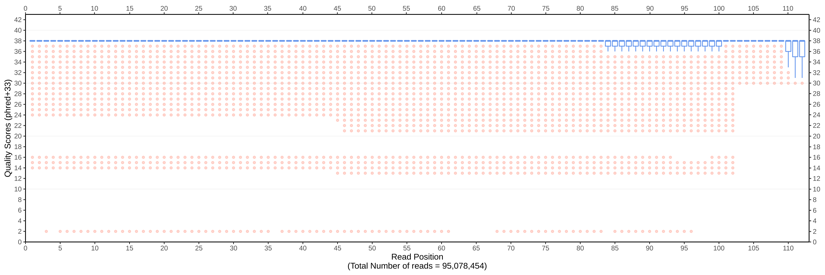

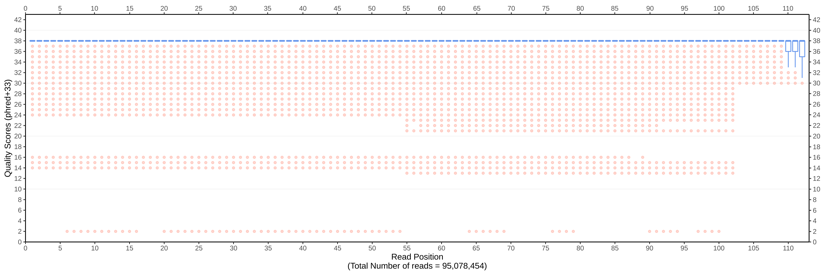


HiSeq 2500 high output (R1) – threshold-filtered

HiSeq 2500 high output (R2) – threshold-filtered


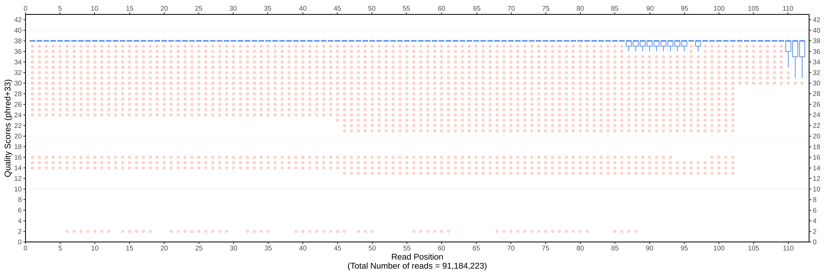

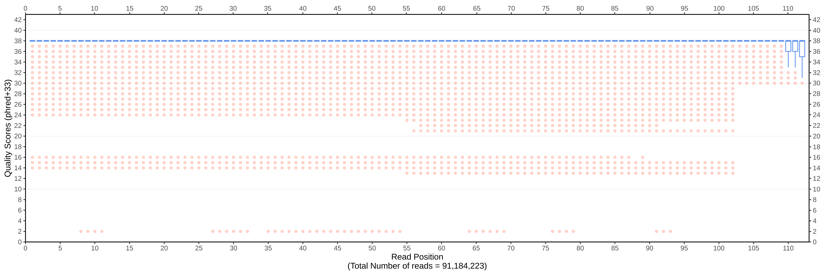


**Figure S2**. Summary (boxplot) of quality scores (Q score) of HiSeq 2500 high output paired end reads at each step of the ngsComposer pipeline. Demultiplexing was performed with a maximum mismatch of 1, while the Q score threshold filtering only allowed for reads with at least 90 % of bases in read having a minimum Q score of 30. Red dots indicate outliers, which also indicates the level of quality score binning.

HiSeq 2500 rapid run (R1) – raw

HiSeq 2500 rapid run (R2) – raw


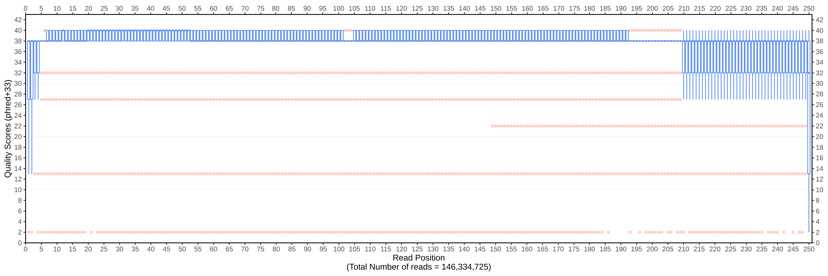

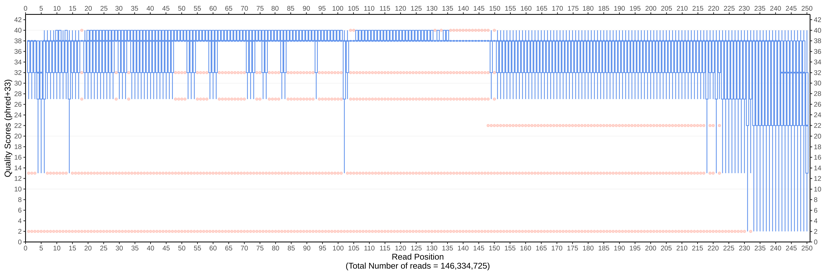


HiSeq 2500 rapid run (R1) – demultiplexed

HiSeq 2500 rapid run (R2) – demultiplexed


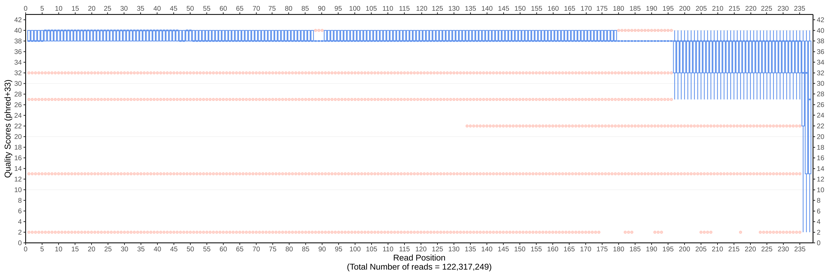

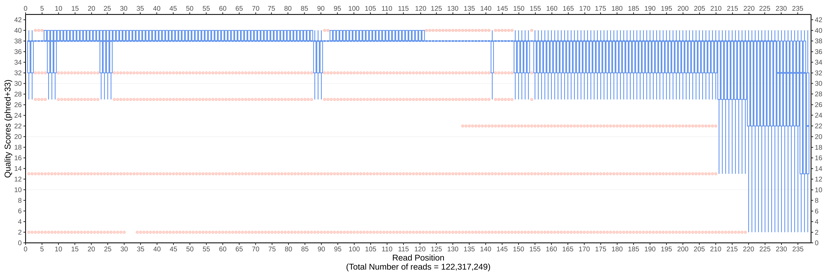


HiSeq 2500 rapid run (R1) – motif-filtered

HiSeq 2500 rapid run (R2) – motif-filtered


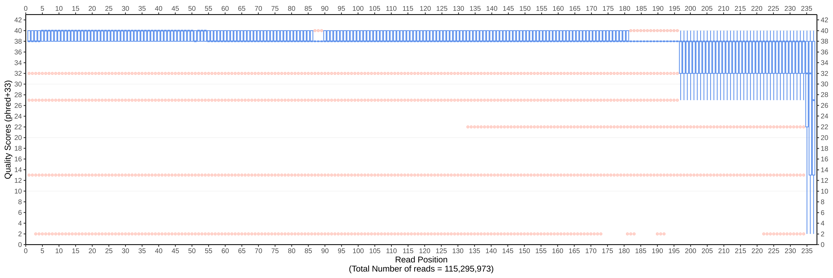

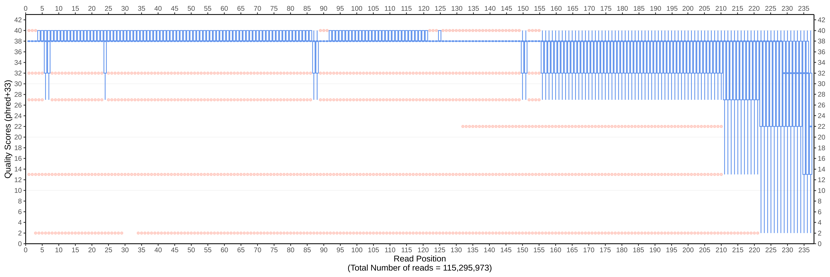


HiSeq 2500 rapid run (R1) – end-trimmed

HiSeq 2500 rapid run (R2) – end-trimmed


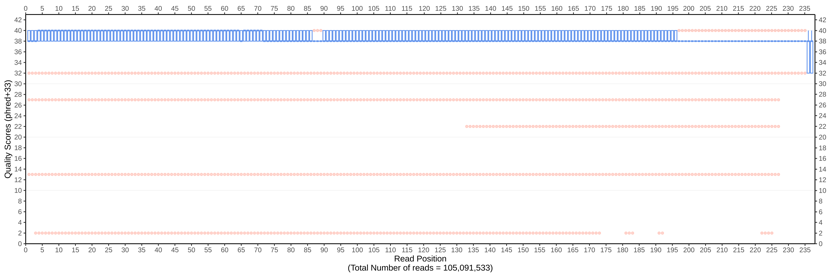

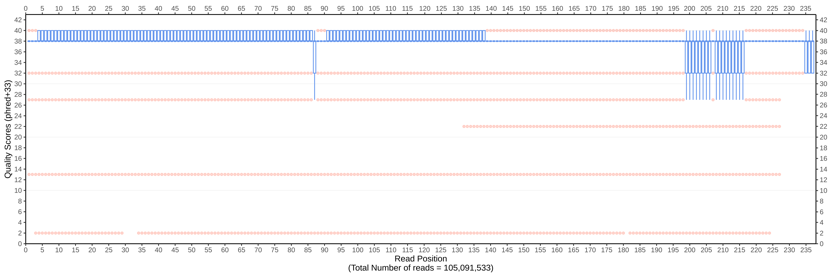


HiSeq 2500 rapid run (R1) – adapted

HiSeq 2500 rapid run (R2) – adapted


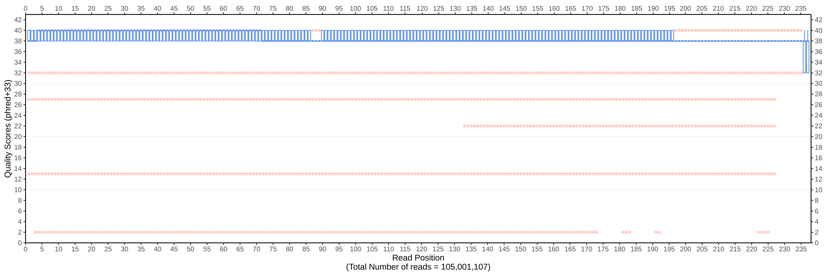

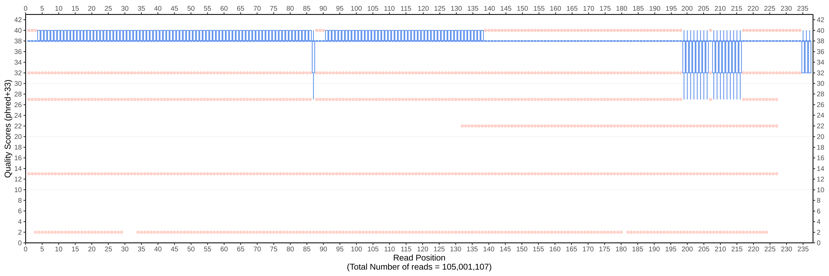


HiSeq 2500 rapid run (R1) – threshold-filtered

HiSeq 2500 rapid run (R2) – threshold-filtered


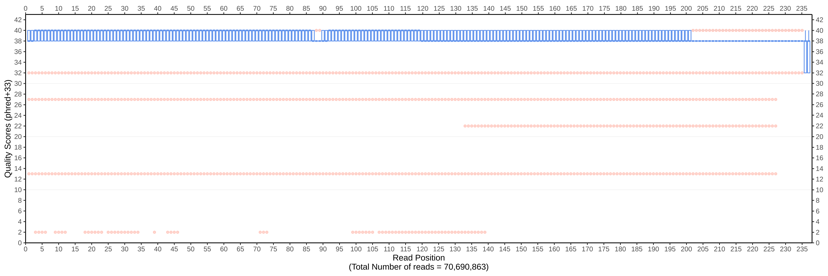

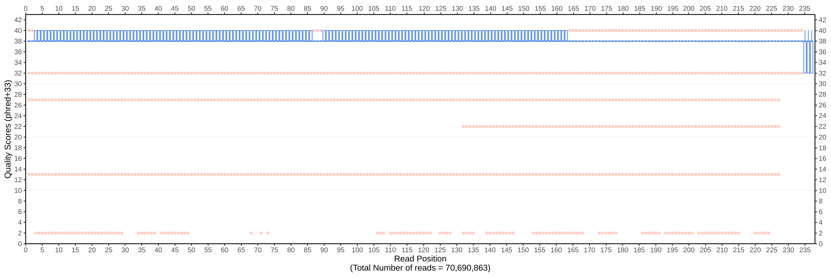


**Figure S3**. Summary (boxplot) of quality scores (Q score) of HiSeq 2500 rapid run paired end reads at each step of the ngsComposer pipeline. Demultiplexing was performed with a maximum mismatch of 1, while the Q score threshold filtering only allowed for reads with at least 90 % of bases in read having a minimum Q score of 30. Red dots indicate outliers, which also indicates the level of quality score binning.

NovaSeq (R1) – raw

NovaSeq (R2) – raw


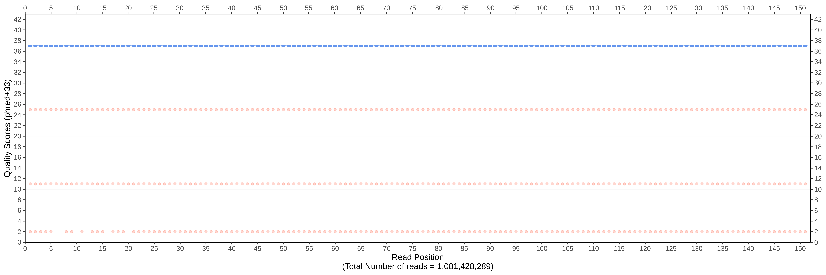

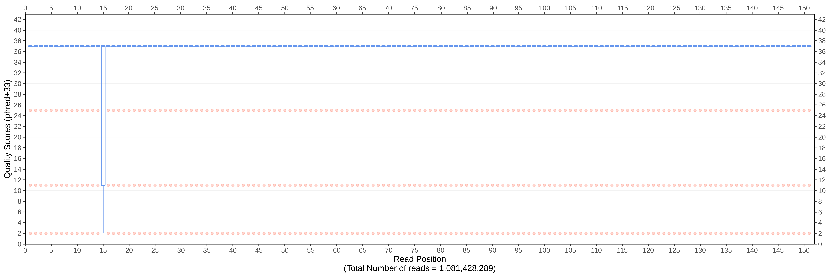


NovaSeq (R1) – demultiplexed

NovaSeq (R2) – demultiplexed


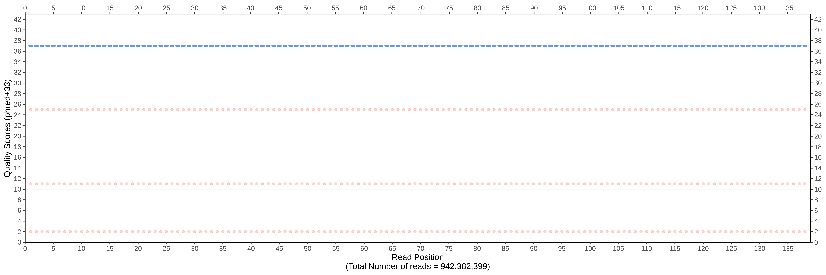

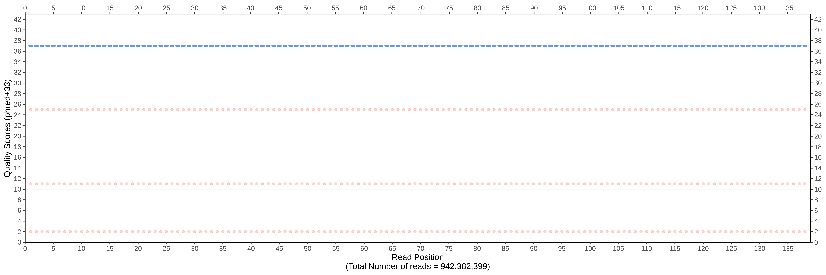


NovaSeq (R1) – motif-filtered

NovaSeq (R2) – motif-filtered


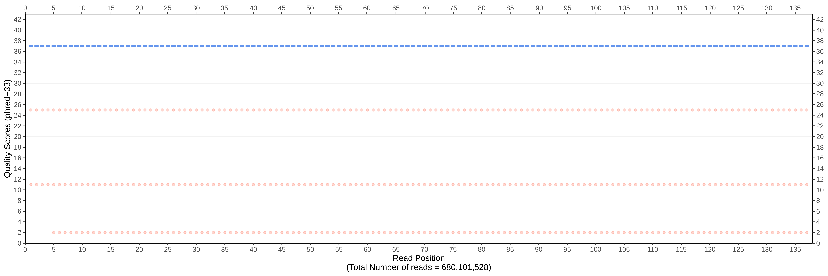

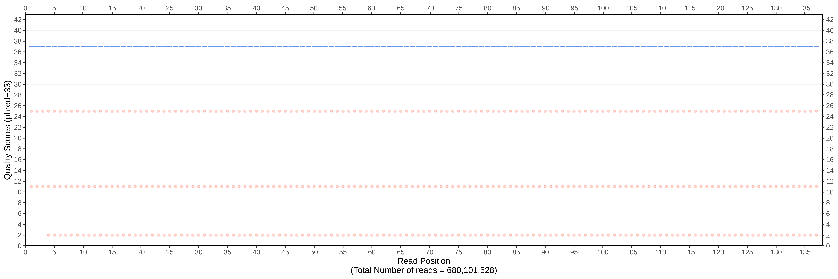


NovaSeq (R1) – end-trimmed

NovaSeq (R2) – end-trimmed


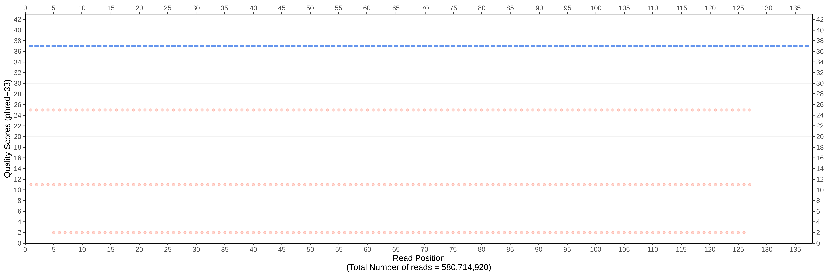

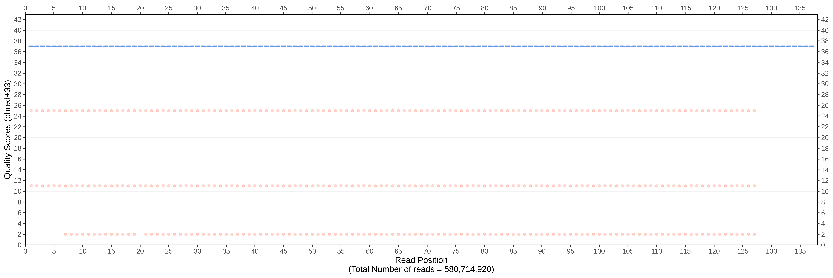


NovaSeq (R1) – adapted

NovaSeq (R2) – adapted


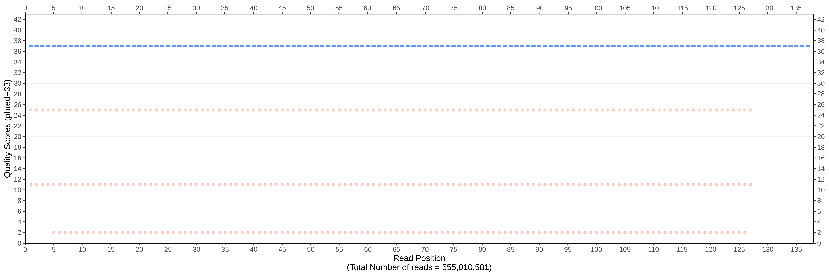

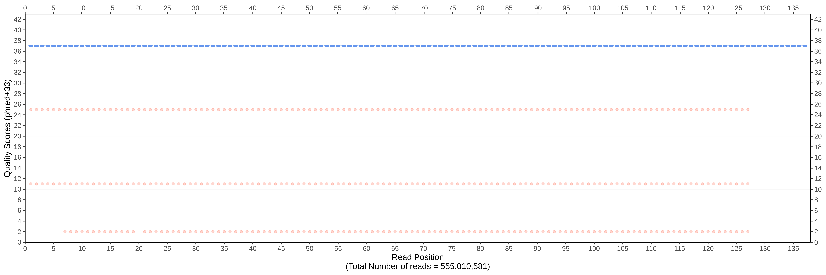


NovaSeq (R1) – threshold-filtered

NovaSeq (R2) – threshold-filtered


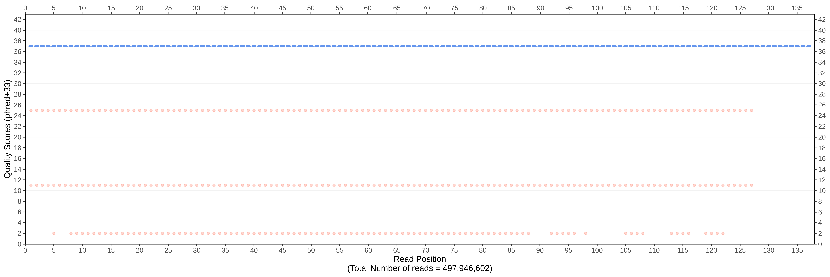

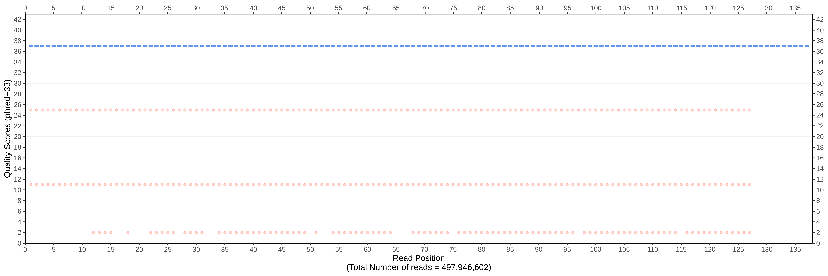


**Figure S4**. Summary (boxplot) of quality scores (Q score) of NovaSeq 6000 paired end reads at each step of the ngsComposer pipeline. Demultiplexing was performed with a maximum mismatch of 1, while the Q score threshold filtering only allowed for reads with at least 90 % of bases in read having a minimum Q score of 30. Red dots indicate outliers, which also indicates the level of quality score binning.


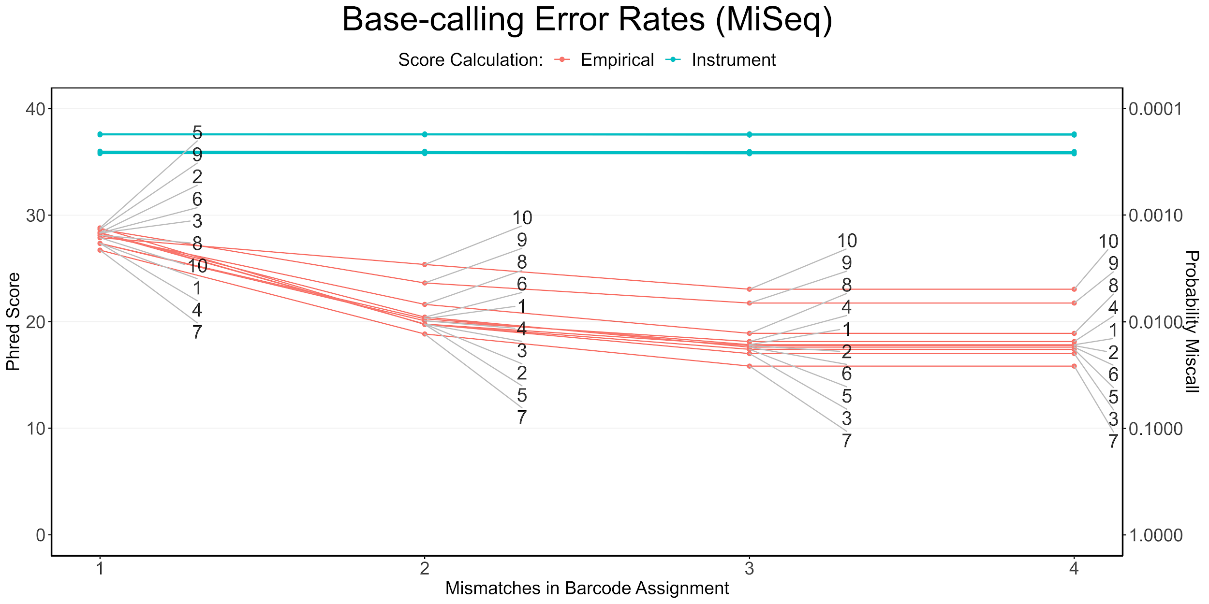


**Figure S5**. Comparison of instrument-derived (blue) and Empirically calculated (red) Q Scores from bases in the barcode region of MiSeq R1 reads. The position specific contribution of the barcode region is labelled at Hamming distances of 1 through 4. The scores plateau around the lenient Hamming distance of 3 as the demultiplexing can’t assign a barcode when multiple sample assignments are possible.


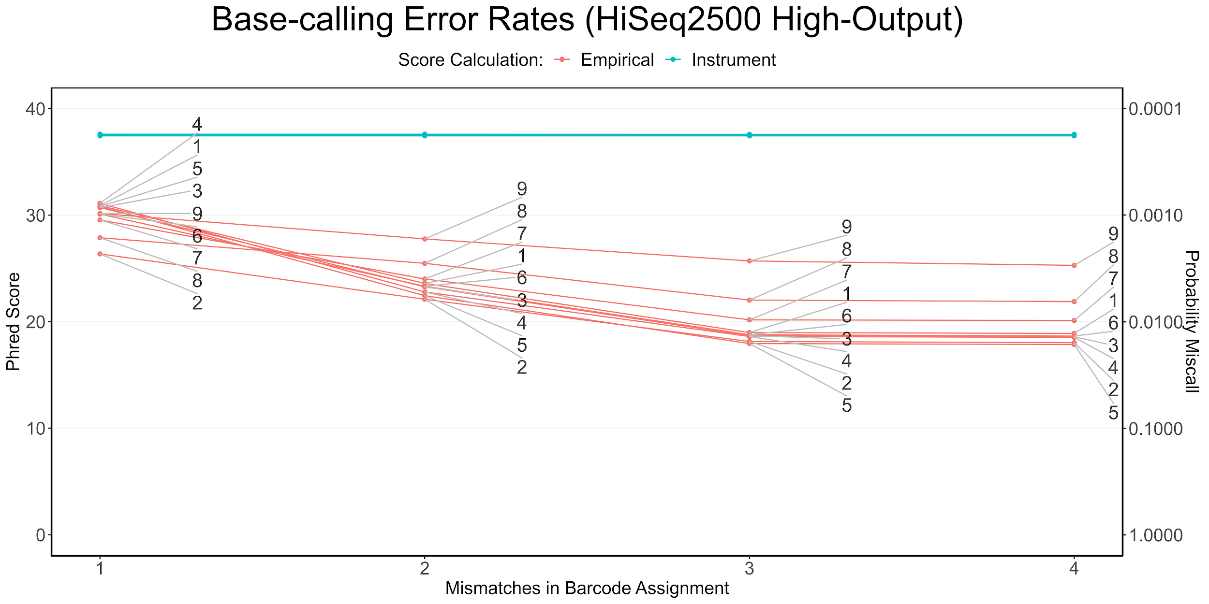


**Figure S6**. Comparison of instrument-derived (blue) and Empirically calculated (red) Q Scores from bases in the barcode region of HiSeq 2500 High Output R1 reads. The position specific contribution of the barcode region is labelled at Hamming distances of 1 through 4. The scores plateau around the lenient Hamming distance of 3 as the demultiplexing can’t assign a barcode when multiple sample assignments are possible.


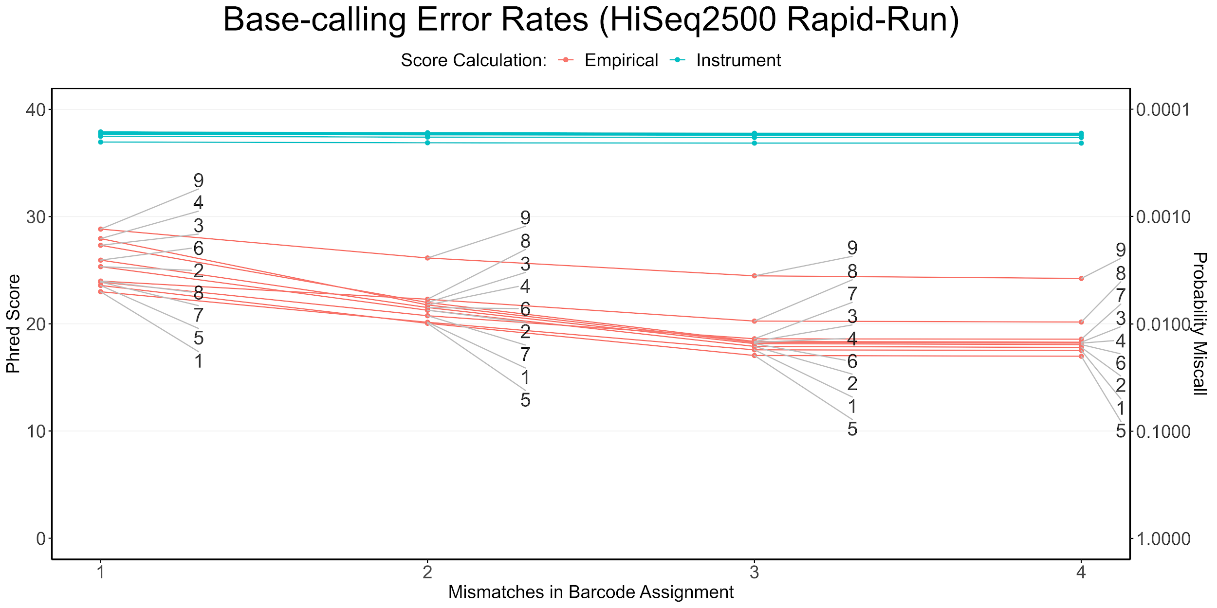


**Figure S7**. Comparison of instrument-derived (blue) and Empirically calculated (red) Q Scores from bases in the barcode region of HiSeq 2500 Rapid Run R1 reads. The position specific contribution of the barcode region is labelled at Hamming distances of 1 through 4. The scores plateau around the lenient Hamming distance of 3 as the demultiplexing can’t assign a barcode when multiple sample assignments are possible.


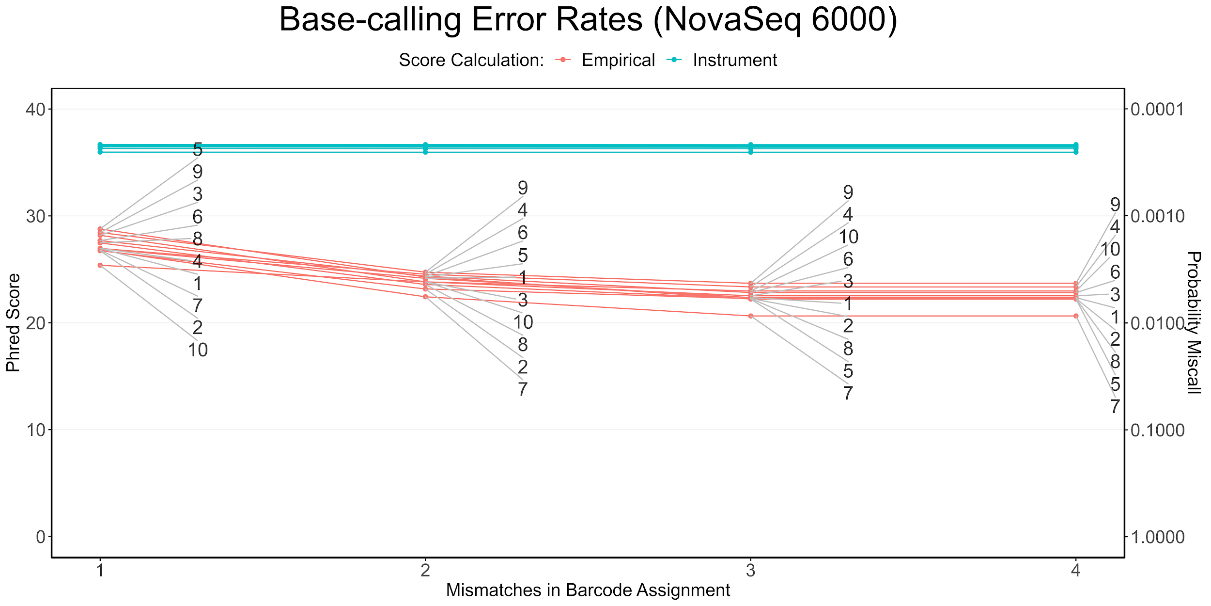


**Figure S8**. Comparison of instrument-derived (blue) and Empirically calculated (red) Q Scores from bases in the barcode region of NovaSeq R1 reads. The position specific contribution of the barcode region is labelled at Hamming distances of 1 through 4. The scores plateau around the lenient Hamming distance of 3 as the demultiplexing can’t assign a barcode when multiple sample assignments are possible.


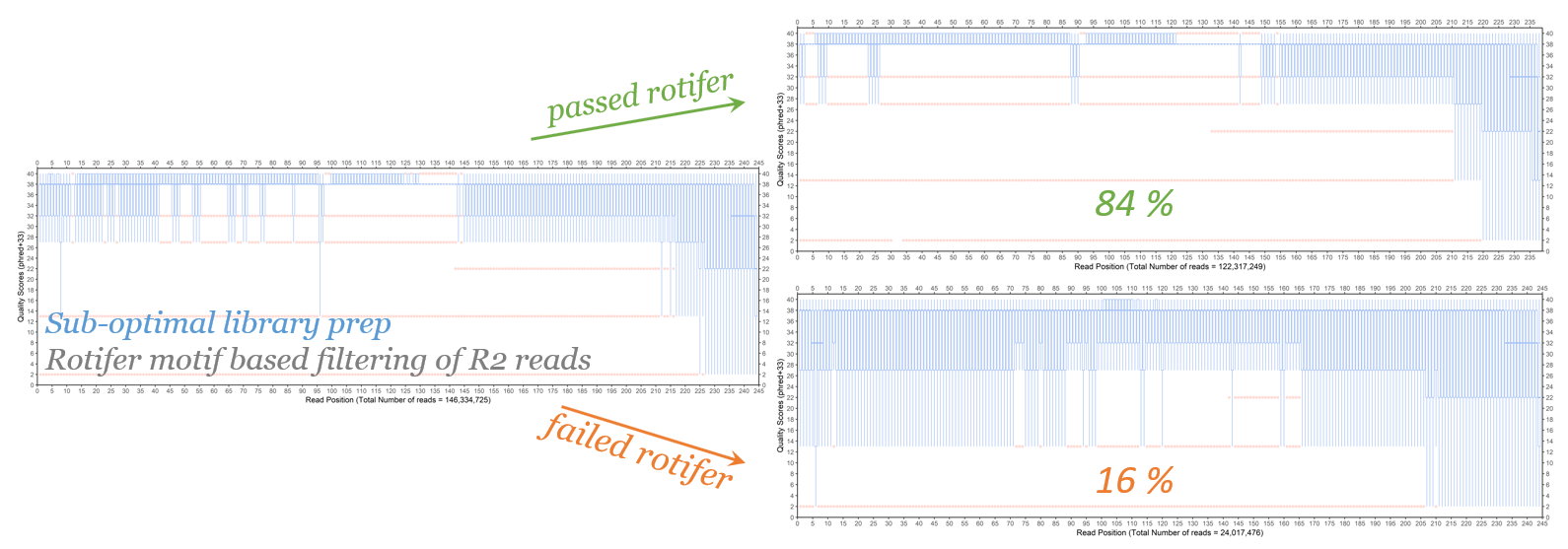


**A**


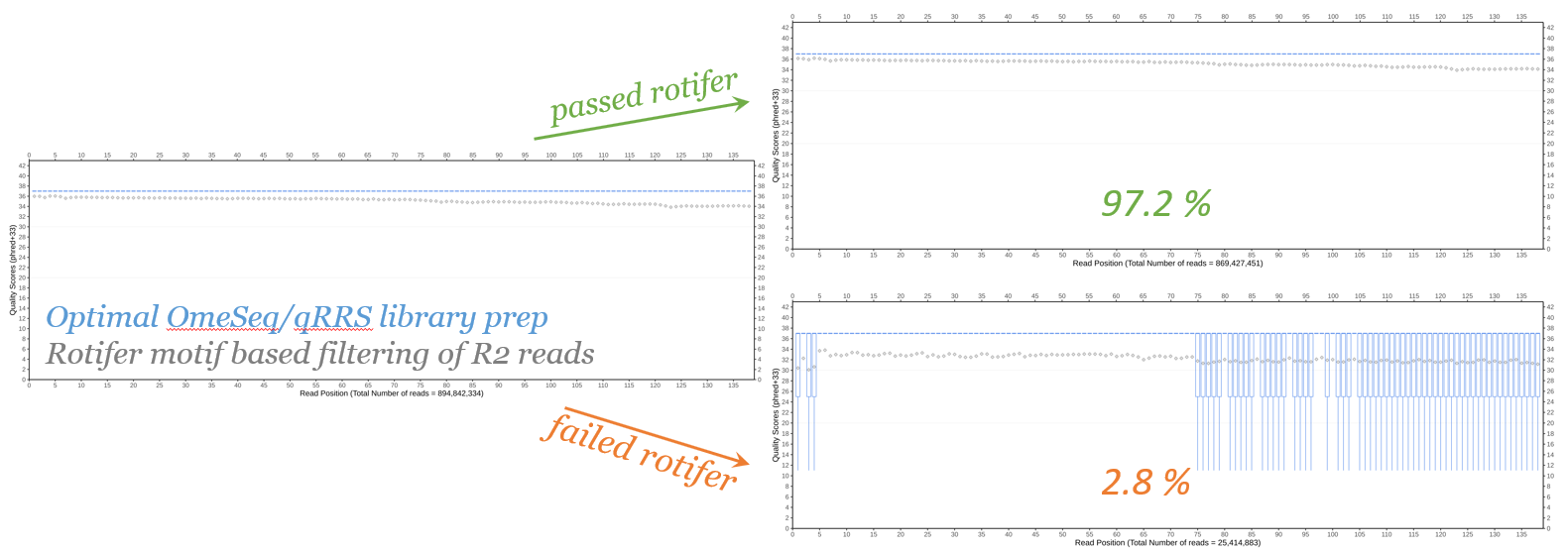


**B**

**Figure S9**. QC plots of low-quality (A: HiSeq 2500 rapid run) and high-quality (B: NovaSeq 6000 SP) sequence dataset of R2 reads. Motif-based error detection and removal algorithm implemented in rotifer (a tool in the ngsComposer pipeline). Reads that fail filtering by rotifer (i.e. using the restriction site motif at beginning of each read) revealed propagation of base calling error along entire length of reads.

R1: unfiltered

R1: failed motif filtering (tool: rotifer) rotifer)


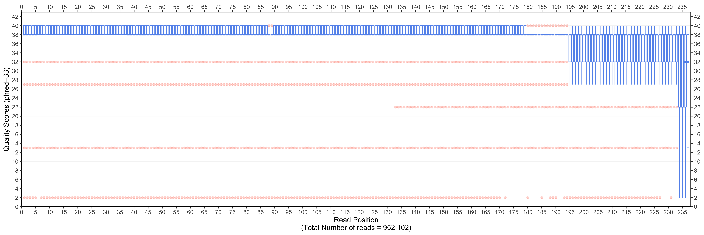

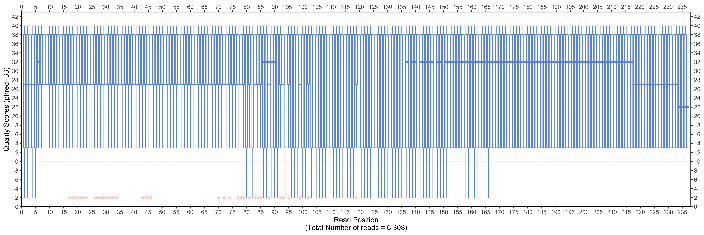


R2: failed motif filtering (tool: rotifer) rotifer)

R2: unfiltered


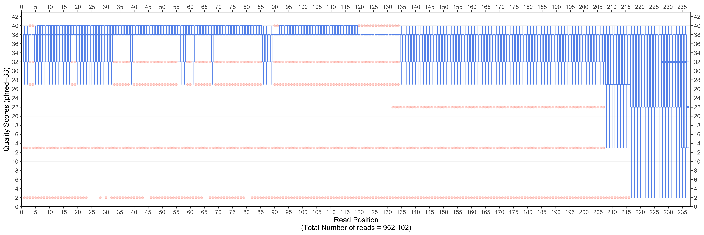

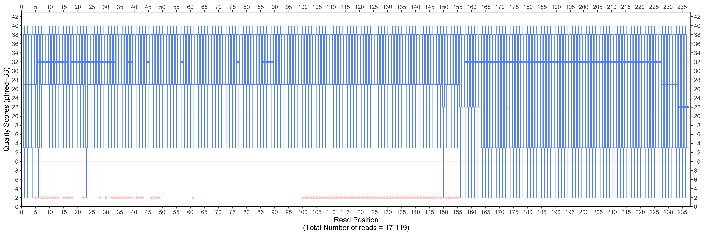


**Figure S10.** Summary (boxplot) of quality scores (Q score) of simulated HiSeq 2500 rapid run paired end reads comparing the reads before and after motif-filtering (rotifer). QC profile of reads that fail motif-filtering (mutated restriction enzyme site sequence) are confirmed to contain low Q scores propagated along the entire length of reads (QC plots on left).


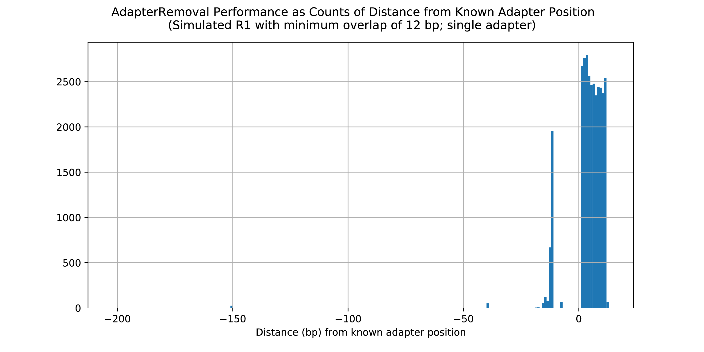

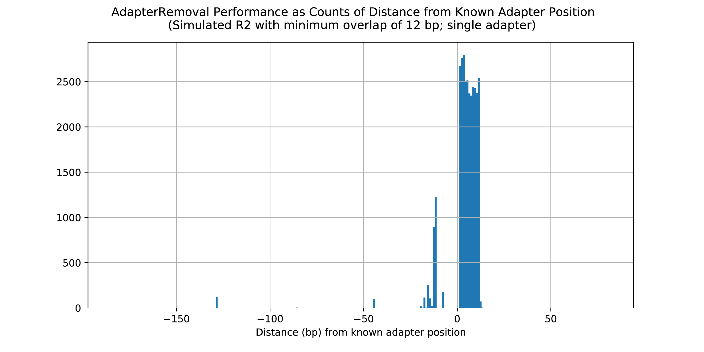


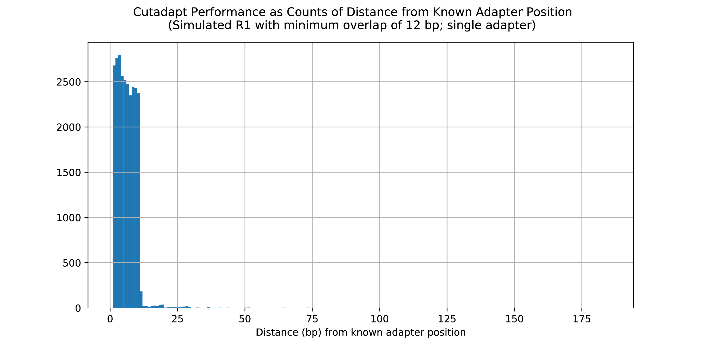

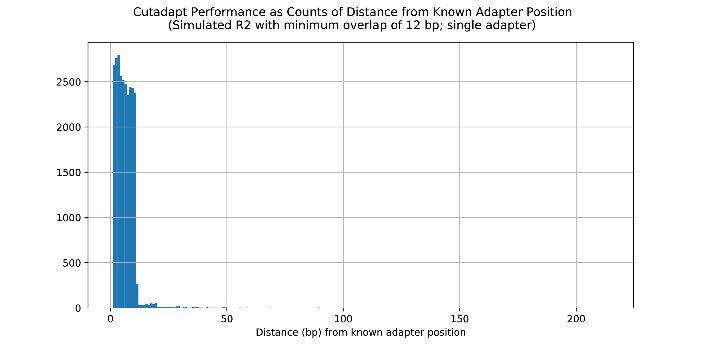


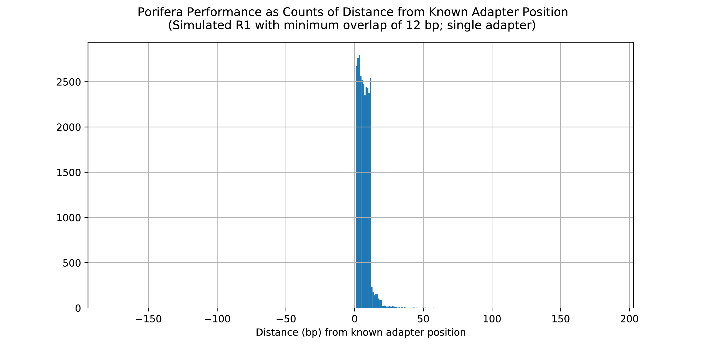

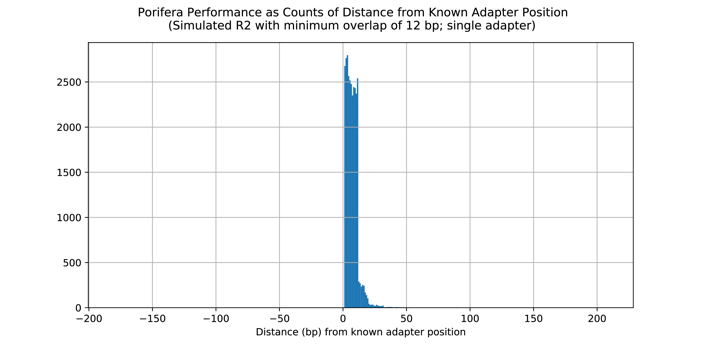


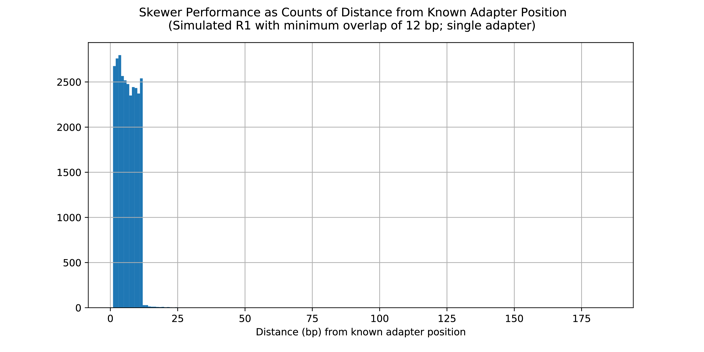

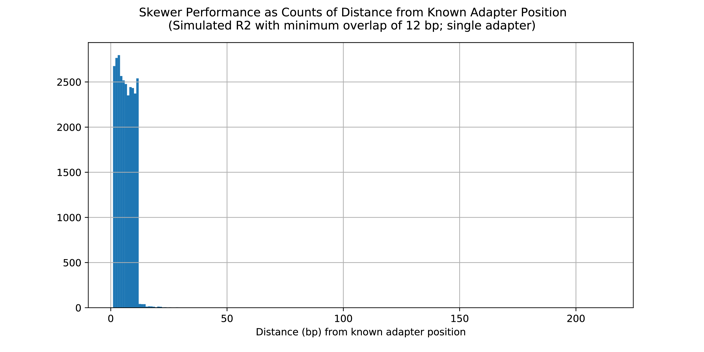


**Figure S11.** Distribution of distances from known adapter position in base pairs (bp) across several common adapter trimming tools using a single, common adapter on the R1and R2 reads of simulated Hiseq 2500 rapid run data. Negative distances are false positives that remove too many bases whereas positive distances are missed adapter regions.


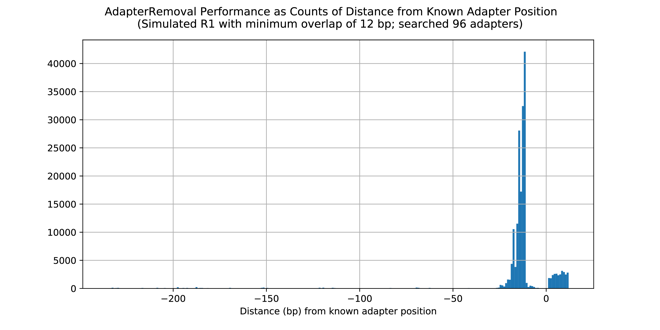

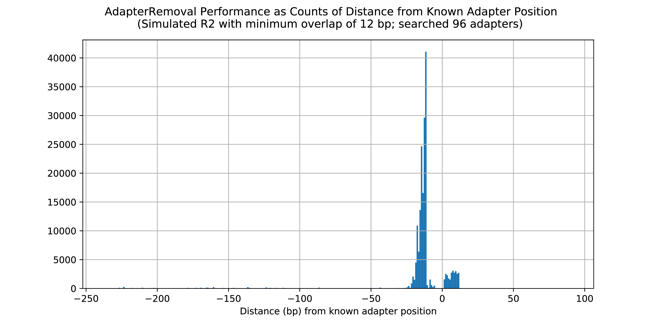


A.


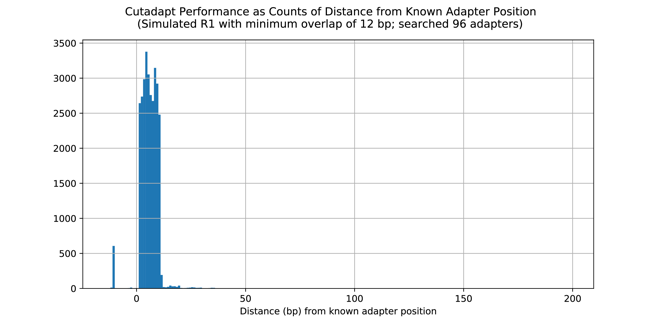

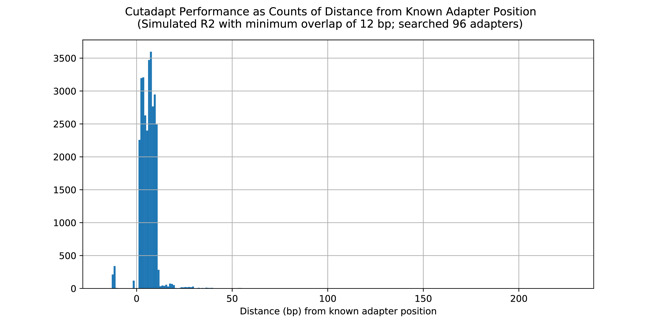


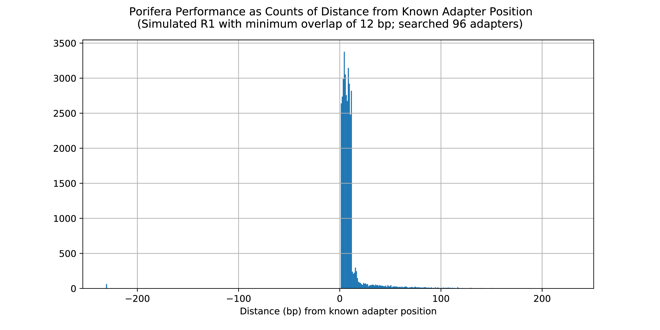

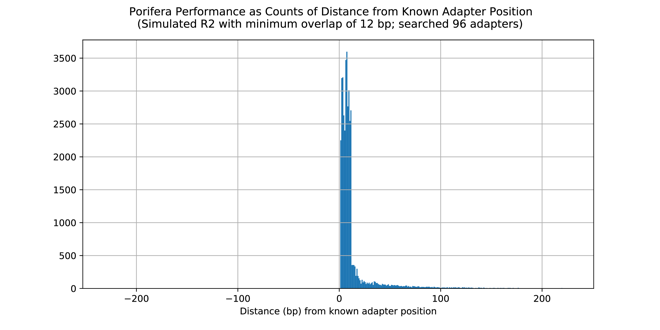


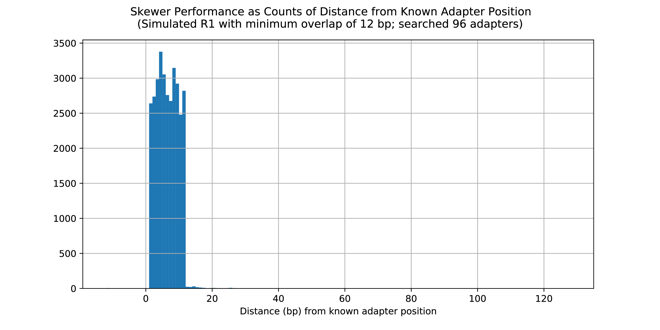

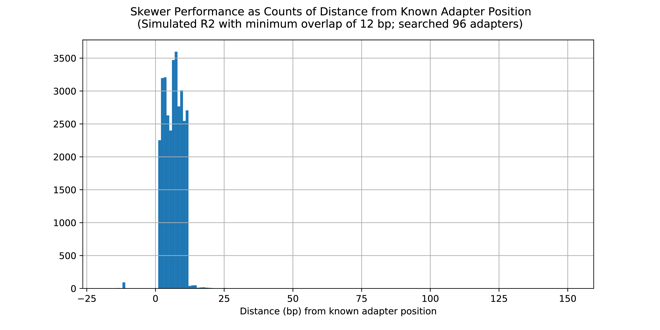


B.


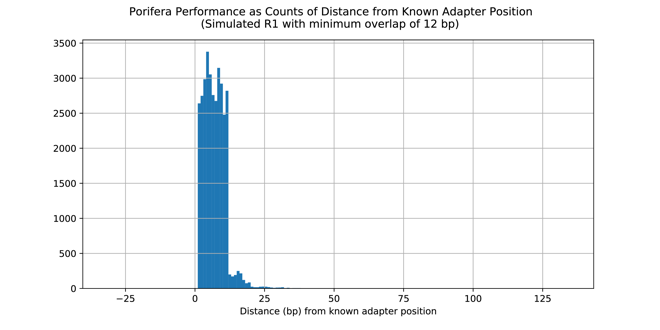

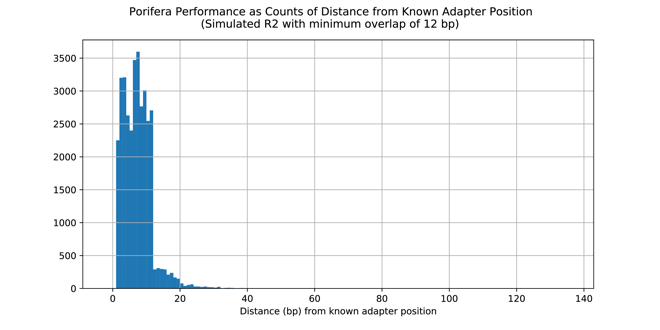


**Figure S12.** Distribution of distances from known adapter position in base pairs (bp) across several common adapter trimming tools using 96 variable length barcoded adapters on the R1and R2 reads of simulated Hiseq 2500 rapid run data. Negative distances are false positives that remove too many bases whereas positive distances are missed adapter regions. Performance of porifera in shown in standalone (A) and in the ngsComposer pipeline mode (B).

C.

**Table S1.** Comparison of empirical and instrument-derived error rates.

| Dataset | Hamming Distance | Mean Q score | Mean empirical score | Q score difference | Fold change in error rate |
| --- | --- | --- | --- | --- | --- |
| HiSeq2500 High-Output | 1 | 37.54 | 29.74 | 7.81 | 6.03 |
| HiSeq2500 High-Output | 2 | 37.54 | 23.87 | 13.67 | 23.29 |
| HiSeq2500 High-Output | 3 | 37.53 | 19.89 | 17.64 | 58.06 |
| HiSeq2500 High-Output | 4 | 37.53 | 19.76 | 17.77 | 59.83 |
| HiSeq2500 Rapid-Run | 1 | 37.67 | 25.54 | 12.13 | 16.33 |
| HiSeq2500 Rapid-Run | 2 | 37.60 | 21.77 | 15.83 | 38.29 |
| HiSeq2500 Rapid-Run | 3 | 37.57 | 18.96 | 18.61 | 72.58 |
| HiSeq2500 Rapid-Run | 4 | 37.57 | 18.87 | 18.70 | 74.10 |
| MiSeq | 1 | 36.55 | 27.99 | 8.56 | 7.18 |
| MiSeq | 2 | 36.55 | 20.96 | 15.59 | 36.26 |
| MiSeq | 3 | 36.54 | 18.53 | 18.01 | 63.22 |
| MiSeq | 4 | 36.54 | 18.53 | 18.01 | 63.25 |
| NovaSeq 6000 | 1 | 36.45 | 27.34 | 9.11 | 8.14 |
| NovaSeq 6000 | 2 | 36.43 | 23.89 | 12.54 | 17.97 |
| NovaSeq 6000 | 3 | 36.43 | 22.51 | 13.92 | 24.65 |
| NovaSeq 6000 | 4 | 36.43 | 22.51 | 13.92 | 24.66 |
